# Sonic Hedgehog activates prostaglandin signaling to stabilize primary cilium length

**DOI:** 10.1083/jcb.202306002

**Published:** 2024-06-10

**Authors:** Shariq S. Ansari, Miriam E. Dillard, Yan Zhang, Mary Ashley Austria, Naoko Boatwright, Elaine L. Shelton, Daniel P. Stewart, Amanda Johnson, Christina E. Wang, Brandon M. Young, Zoran Rankovic, Baranda S. Hansen, Shondra M. Pruett-Miller, Alexandre F. Carisey, John D. Schuetz, Camenzind G. Robinson, Stacey K. Ogden

**Affiliations:** 1 Department of Cell and Molecular Biology, St. Jude Children’s Research Hospital, Memphis, TN, USA; 2 Rhodes College Summer Plus Program, Memphis, TN, USA; 3 Department of Pediatrics, Monroe Carell Jr. Children’s Hospital at Vanderbilt and Vanderbilt University Medical Center, Vanderbilt University School of Medicine, Nashville, TN, USA; 4 Department of Pharmacology, Vanderbilt University School of Medicine, Nashville, TN, USA; 5 Cell and Tissue Imaging Center, St. Jude Children’s Research Hospital, Memphis, TN, USA; 6 Graduate School of Biomedical Sciences, St. Jude Children’s Research Hospital, Memphis, TN, USA; 7 Department of Chemical Biology and Therapeutics, St. Jude Children’s Research Hospital, Memphis, TN, USA; 8 Center for Advanced Genome Engineering, St. Jude Children’s Research Hospital, Memphis, TN, USA; 9 Department of Pharmaceutical Sciences, St. Jude Children’s Research Hospital, Memphis, TN, USA

## Abstract

Sonic Hedgehog (SHH) is a driver of embryonic patterning that, when corrupted, triggers developmental disorders and cancers. SHH effector responses are organized through primary cilia (PC) that grow and retract with the cell cycle and in response to extracellular cues. Disruption of PC homeostasis corrupts SHH regulation, placing significant pressure on the pathway to maintain ciliary fitness. Mechanisms by which ciliary robustness is ensured in SHH-stimulated cells are not yet known. Herein, we reveal a crosstalk circuit induced by SHH activation of Phospholipase A_2_α that drives ciliary E-type prostanoid receptor 4 (EP_4_) signaling to ensure PC function and stabilize ciliary length. We demonstrate that blockade of SHH-EP_4_ crosstalk destabilizes PC cyclic AMP (cAMP) equilibrium, slows ciliary transport, reduces ciliary length, and attenuates SHH pathway induction. Accordingly, *Ep4*^*−/−*^ mice display shortened neuroepithelial PC and altered SHH-dependent neuronal cell fate specification. Thus, SHH initiates coordination between distinct ciliary receptors to maintain PC function and length homeostasis for robust downstream signaling.

## Teaser

This study reveals crosstalk between Sonic Hedgehog (SHH) and prostaglandin signaling pathways. SHH signaling leads to the secretion of PGE2, which activates the ciliary EP4 receptor to promote primary cilium length stability. Disruption of SHH-to-EP4 communication leads to primary cilium shortening and reduced SHH signal output.

## Introduction

During embryonic patterning, Sonic Hedgehog (SHH) family signaling proteins provide instructional cues that guide cell fate decisions to organize tissues. Disruption of SHH pathway activation leads to developmental disorders, and aberrant SHH activity drives cancer, underscoring the importance of tight signal control (Barakat et al., 2010). Accordingly, sophisticated regulatory mechanisms are in place to ensure appropriate pathway activity levels in both basal and activated signaling states. Pathway regulation requires localization of SHH signaling components to a specialized sensory organelle called the primary cilium (plural: primary cilia, both referred to as PC) (Caspary et al., 2007; Huangfu and Anderson, 2006). Each cell possesses a single PC that is anchored to the basal body and allows for the organization of numerous signal transduction cascades that instruct diverse cellular responses (Anvarian et al., 2019; Wheway et al., 2018). Despite the PC membrane being contiguous with the plasma membrane, it maintains a distinct membrane lipid composition through the action of lipid metabolic enzymes that localize near the ciliary base (Arensdorf et al., 2017; Findakly et al., 2021; Garcia et al., 2018; Raleigh et al., 2018). Further, a cytoplasmic diffusion barrier controls the entry of soluble proteins into the ciliary body, ensuring that the PC offers a protected environment for the interpretation and transduction of myriad extracellular signals (Garcia et al., 2018; Shi et al., 2017; Ye et al., 2013).

The SHH receptor Patched (PTCH), signal transducer Smoothened (SMO), and GLI2/GLI3 transcriptional effectors all function at or cycle through the PC in ligand-regulated manners (Arensdorf et al., 2016; Petrov et al., 2017). In the absence of SHH, SMO occupancy in the PC is blocked by PTCH-mediated depletion of SMO-activating sterols from the ciliary membrane (Kinnebrew et al., 2019, 2021; Kong et al., 2019; Myers et al., 2017; Zhang et al., 2018). In this off state, phosphorylation of GLI2 and GLI3 transcription factors by cyclic AMP (cAMP)-dependent protein kinase (PKA) tags them for partial degradation to remove their transcriptional activation domains (Bangs and Anderson, 2017; Haycraft et al., 2005; Tuson et al., 2011). SHH binding to PTCH attenuates PTCH-mediated ciliary sterol elimination. This leads to SMO-sterol binding, which promotes its accumulation in PC, where it signals to block PKA-promoted GLI truncation. This allows for the accumulation of full-length GLI2/3 proteins, which are subsequently activated for nuclear translocation and target gene induction (Niewiadomski et al., 2014; Umberger and Ogden, 2021).

Current models suggest the dependency of SHH signaling on PC evolved due to the specialized lipid composition of the ciliary membrane allowing for tight control over the availability of SMO-activating sterols (Kinnebrew et al., 2019, 2021; Kong et al., 2019; Raleigh et al., 2018). The small size of the PC allows for rapid modulation of second messengers including cAMP (Hansen et al., 2020; Truong et al., 2021). SHH signal output is highly sensitive to changes in ciliary cAMP concentration due to the key role that PKA plays in the regulation of PC-localized GLI (Mukhopadhyay et al., 2013; Truong et al., 2021; Vuolo et al., 2015). Accordingly, G protein-coupled receptor (GPCR) signaling can influence SHH pathway activity in both basal and activated states. In the absence of SHH, the Gα_s_-coupled PC-localized GPCR GPR161 raises ciliary cAMP by stimulating ciliary adenylyl cyclases (AC). Pathway induction lowers ciliary cAMP by stimulating GPR161 ciliary exit (Mukhopadhyay et al., 2013). In addition, SMO can activate Gα_i_-coupled heterotrimeric G proteins, which directly inhibit AC to pause cAMP production (Barzi et al., 2011; Ogden et al., 2008; Riobo and Manning, 2007). Thus, there are at least two direct routes by which SHH can lower ciliary cAMP to reduce PKA-directed GLI processing. How SHH-mediated reduction of PC cAMP impacts ciliary homeostasis or signaling by other PC-localized GPCRs has not yet been described.

A growing body of evidence supports that ciliary function and length stability require high ciliary cAMP. Studies in zebrafish demonstrate that multidrug resistance protein 4 transporter (MRP4/ABCC4), one of the MRPs that export the prostaglandin signaling molecule PGE_2_, is required for motile cilium elongation and length stability (Cole, 2014). PGE_2_ activates the Gα_s_-coupled ciliary GPCR E-type prostanoid receptor 4 (EP_4_), which raises intraciliary cAMP levels to promote anterograde intraflagellar transport (IFT) and maintain motile cilium length (Besschetnova et al., 2010; Jin et al., 2014; Marra et al., 2019). PC length control likely occurs through similar mechanisms because ABCC4 knockdown in cultured fibroblasts leads to PC shortening (Jin et al., 2014). This raises the possibility that ciliary cAMP reduction occurring in response to high-level activation of SHH signaling could slow IFT, shorten PC, and blunt the downstream signal response. The fail-safes that prevent ciliary shortening in SHH-stimulated cells are not known.

We previously reported that SHH activates cytosolic phospholipase A_2_α (cPLA_2_α) to produce arachidonic acid, which drives a feed-forward signal that enhances SMO ciliary enrichment. Inhibition of cPLA_2_α attenuates agonist-induced SMO ciliary accumulation and blunts signaling to GLI transcription factors (Arensdorf et al., 2017). Arachidonic acid can be metabolized to generate the EP_4_ agonist PGE_2_, which led us to hypothesize that, in addition to enhancing SMO ciliary enrichment, SHH-mediated production of arachidonic acid may connect the SHH pathway with PGE_2_/EP_4_-regulated PC length control. We tested this hypothesis and mapped a communication relay between the SHH and prostaglandin signaling pathways that equilibrates ciliary cAMP for PC length homeostasis and SHH signaling competency. We demonstrate that genetic ablation or small-molecule inhibition of SHH-to-EP_4_ crosstalk destabilizes PC cAMP levels, slows IFT, shortens PC, reduces SMO ciliary enrichment, and attenuates SMO signaling to GLI. Consistent with EP_4_ signaling contributing to SHH pathway activation in vivo, mice lacking EP_4_ have shortened neuroepithelial PC and exhibit neural tube patterning defects indicative of compromised SHH signaling. As such, we propose the SHH pathway linked with prostaglandin signaling through cPLA_2_α activation to ensure PC length stability for optimal function of the organelle from which it instructs downstream signaling.

## Results

### cPLA_2_α contributes to ciliary length control downstream of SHH

**Figure 1.**
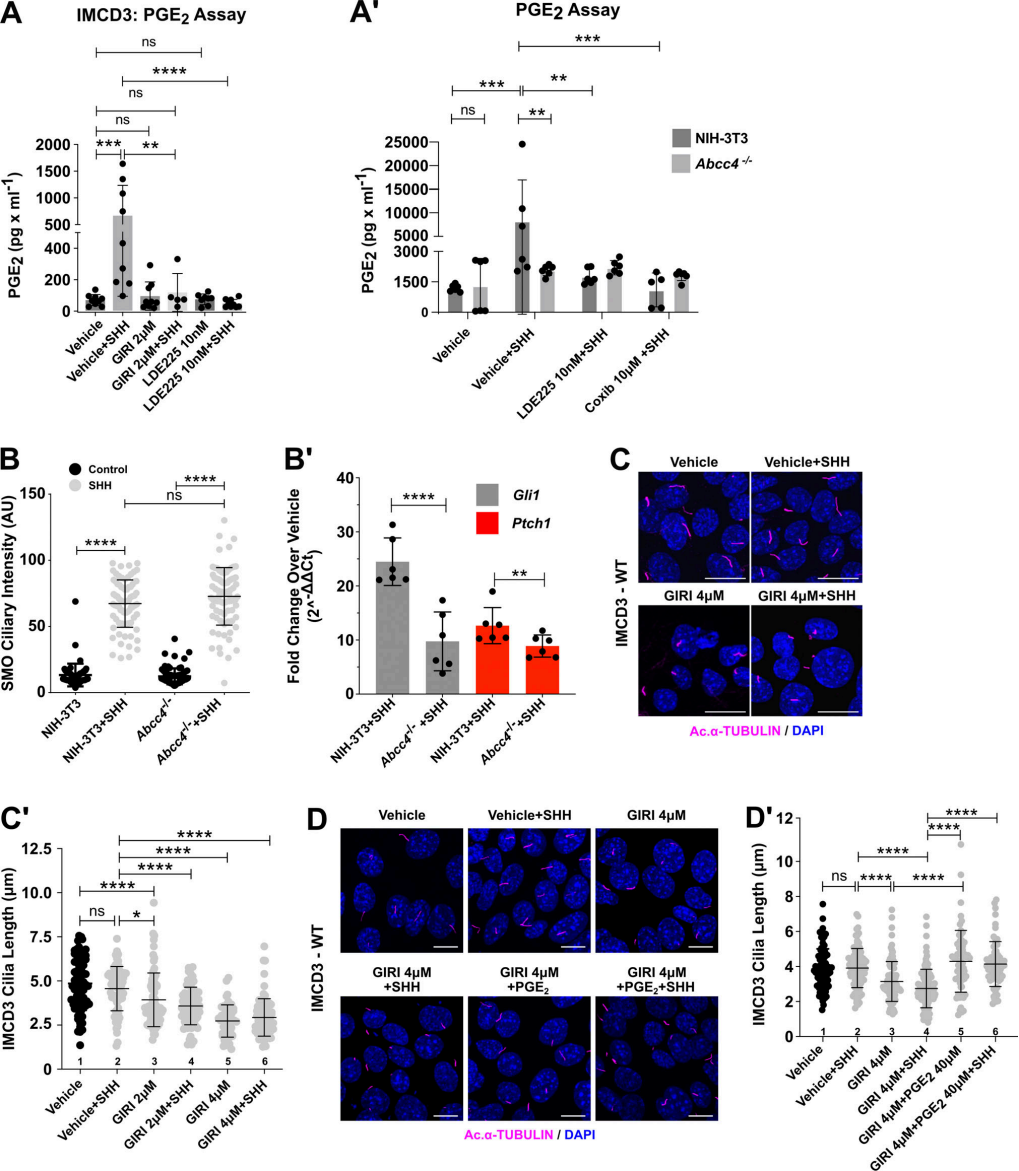
**PGE**_**2**_ **generated downstream of SHH contributes to PC length control. (A and A′)** PGE_2_ was measured in IMCD3 (A) or WT and *Abcc4*^*−/−*^ NIH-3T3 (A′) cell culture media by ELISA. **(A)** Cells were pretreated with vehicle (DMSO), SMO inhibitor LDE225 (10 nM), or cPLA_2_α inhibitor giripladib (GIRI, 2 µM), and then exposed to SHH or control conditioned media. PGE_2_ was measured in cell culture supernatant ∼36 h after treatment. **(A′)** Cells were pretreated with vehicle (DMSO), LDE225 (10 nM), or COX2 inhibitor Celecoxib (Coxib,10 µM) and then stimulated with control or SHH-conditioned media. PGE_2_ was measured in the supernatant ∼36 h after treatment. ELISA experiments were performed at least twice with three biological replicates per experiment. Pooled data are shown. **(B)** SMO PC signal intensity was determined for NIH-3T3 and *Abcc4*^*−/−*^ cells treated with control or SHH-conditioned media. **(B′)** NIH-3T3 and *Abcc4*^*−/−*^ cells were stimulated with control or SHH conditioned media for 18 h and induction of SHH targets *Gli1* and *Ptch1* was determined by qRT-PCR. Fold change over control conditioned media treatment was calculated using the 2^–∆∆Ct^ method. **(C)** IMCD3 cells were treated with SHH-conditioned media in the presence of GIRI (4 µM) or vehicle (DMSO). The PC axoneme is marked by acetylated α-tubulin (magenta). DAPI (blue) marks nuclei. Scale bar = 10 μm. **(C′)** Lengths of PC were quantified in IMCD3 cells treated with SHH-conditioned media in the absence or presence of GIRI. Average PC length was determined by measuring cilia of ≥100 cells/condition across three independent experiments. **(D)** IMCD3 cells were treated with GIRI (4 μM) in the presence of PGE_2_ (40 µM) or vehicle. Scale bar = 10 μm. **(D′)** Quantification is shown in D′. For all experiments, statistical significance was calculated using one-way ANOVA. A P value of <0.05 was considered statistically significant with significance indicated as follows: *<0.05, **<0.01, ***<0.001, ****<0.0001, and ns, P > 0.05. Data are represented as mean ± SD.

We previously reported that Gβγ signaling downstream of SHH-activated SMO stimulates cPLA_2_α to produce arachidonic acid, which drives a feed-forward signal that promotes SMO ciliary accumulation (Arensdorf et al., 2017). To investigate how SHH-mediated cPLA_2_α activation impacts ciliary length homeostasis, we used murine kidney inner medullary collecting duct (IMCD3) cells because they are SHH-responsive, have long PC, and provide an established model for examination of ciliary biology (Breslow et al., 2018; May et al., 2021; Ott and Lippincott-Schwartz, 2012; Zhou et al., 2014). To test whether SHH activation of cPLA_2_α enhanced secretion of the arachidonic acid metabolite PGE_2_, we measured PGE_2_ levels in conditioned media collected from IMCD3 cells stimulated with control conditioned media or conditioned media containing the amino-terminal signaling domain of SHH for ∼36 h. Enzyme-linked immunosorbent assays (ELISA) of conditioned media detected PGE_2_ in the absence of SHH and revealed a significant increase in PGE_2_ levels following SHH exposure. The SHH-stimulated increase in PGE_2_ concentration was blocked by pretreatment with the SMO antagonist LDE225 or the cPLA_2_α-specific inhibitor giripladib (GIRI) (Fig. 1 A) (Arensdorf et al., 2017; Duvernay et al., 2015). Likely due to the activity of cPLA_2_ isoforms that are not inhibited by GIRI, basal PGE_2_ secretion was not reduced following GIRI treatment. As such, we conclude that the increased amount of PGE_2_ that is released following SHH stimulation is produced from cPLA_2_α-generated arachidonic acid.

**Figure S1.**
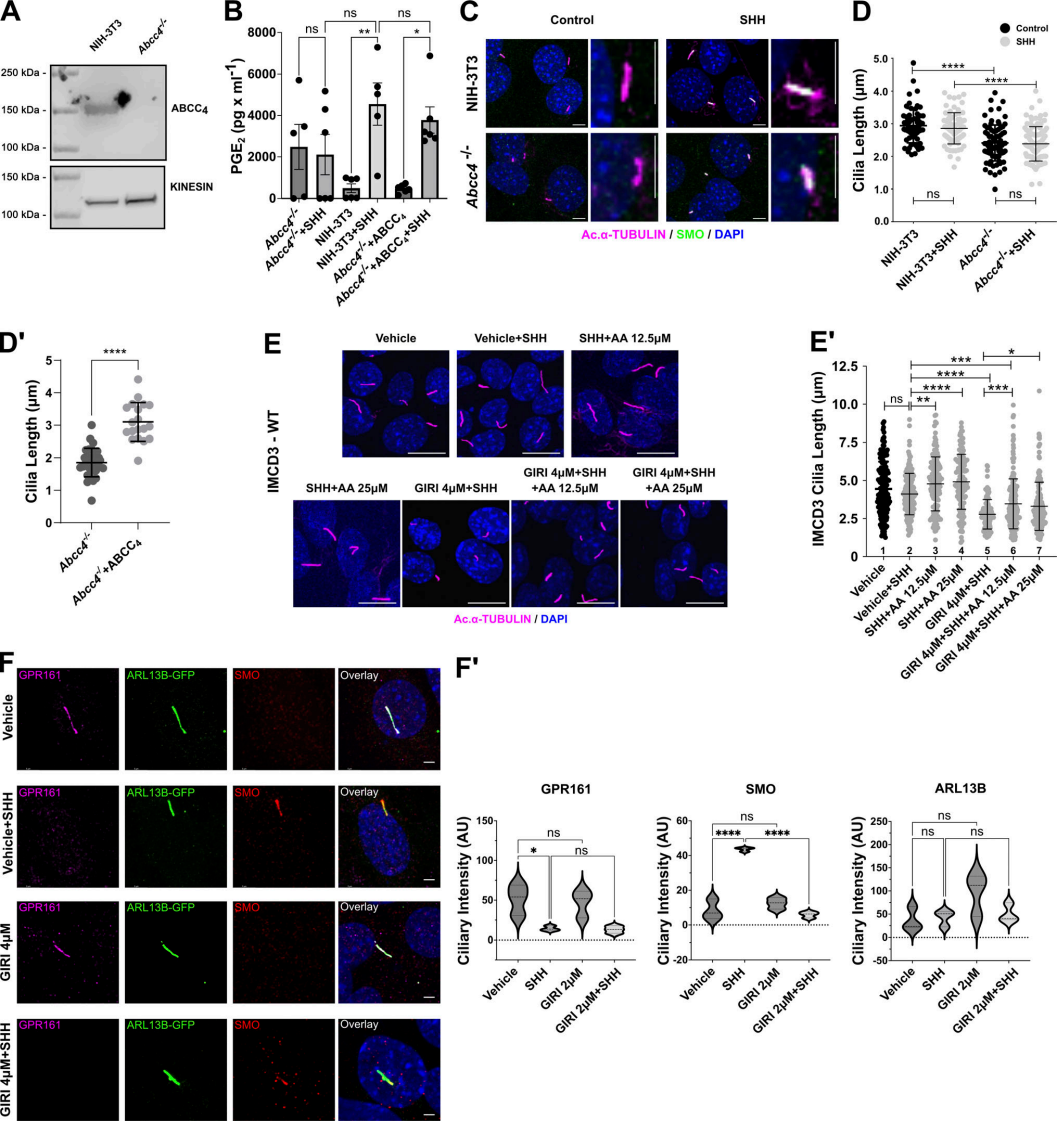
**Further analysis of* Abcc4*^−/−^ cells and arachidonic acid effects on ciliary length and SMO ciliary trafficking. (A)** Western blot of ABCC4 protein in control and *Abcc4*^*−/−*^ NIH-3T3 cells. Kinesin is the loading control. **(B)** Measurement of PGE_2_ in conditioned media of NIH-3T3 control, *Abcc4*^*−/−*^, and *Abcc4*^−/−^ cells transfected with ABCC4 expression vector. PGE_2_ was measured in the supernatant ∼36 h after SHH or control-conditioned media treatment. The experiment was performed twice with three biological replicates per experiment. All data were pooled. For all experiments error bars indicate SD. **(C)** The indicated cell types were treated with control or SHH-conditioned media. The ciliary axoneme is marked by acetylated α-tubulin (magenta) and SMO is shown in green. DAPI (blue) marks nuclei. Scale = 5 μm. **(D and D′)** PC of ≥100 cells/condition was measured to determine the average ciliary length. Experiments were performed at least twice and all data were pooled. Statistical significance was determined using one-way ANOVA in D and a Student’s *t* test in D′. **(E)** IMCD3 cells were treated with control or SHH-conditioned media in the presence of vehicle or 4 µM GIRI plus increasing concentrations of arachidonic acid (12.5 or 25 µM). The ciliary axoneme is marked by acetylated α-tubulin (magenta) and DAPI (blue) marks the nuclei. Scale bar = 10 μm. **(E′)** Ciliary length quantification is shown in E′. **(F)** IMCD3 cells were treated with vehicle or GIRI (4 μM) in the absence or presence of SHH. GPR161 is magenta, SMO is red, ARL13B is green and marks PC. Scale bar = 3 μm. **(F′)** Quantification of ciliary signal intensity for GPR161, SMO, and ARL13B in cells treated with SHH, GIRI, or vehicle control. The experiment was performed twice with 30 cilia imaged per condition per experiment and all data were pooled. For all graphs, significance was determined using a one-way ANOVA. Significance is indicated as follows: *<0.05, **<0.01, ***<0.001, ****<0.0001, and ns, P > 0.05. Data are represented as mean ± SD. Source data are available for this figure: SourceData FS1.

The MRP ABCC4 contributes to PGE_2_ release, ciliary elongation, and SHH signaling activity (Jin et al., 2014; Wijaya et al., 2020). Thus, we tested whether SHH-stimulated PGE_2_ secretion occurred in an ABCC4-dependent manner by analyzing its release from control and SHH-stimulated *Abcc4*^*−/−*^ NIH-3T3 cells (Fig. 1 A′ and Fig. S1 A) (Wijaya et al., 2020). Similar to what was observed with IMCD3 cells, SHH stimulation of control NIH-3T3 cells increased PGE_2_ levels in cell culture media. This increase was blocked by inhibiting SMO or the cyclooxygenase enzyme (COX) that initiates arachidonic acid to PGE_2_ conversion (Fig. 1 A′, Coxib). *Abcc4*^−/−^ cells failed to increase prostaglandin release into media following SHH exposure (Fig. 1 A′). Importantly, PGE_2_ secretion was rescued by expression of exogenous ABCC4 in *Abcc4*^*−/−*^ cells, suggesting that it is the primary MRP transporter responsible for PGE_2_ release following SHH stimulation (Fig. S1 B).

A previous report demonstrated that *Abcc4* knockdown reduced PC length in NIH-3T3 cells (Jin et al., 2014). Thus, we measured the lengths of PC in control and knockout cells and found that the average length of acetylated α-tubulin-labeled PC was significantly reduced in *Abcc4*^*−/−*^ cells compared with control NIH-3T3 fibroblasts (Fig. S1, C and D). Average PC length was restored by ABCC4 reintroduction, suggesting that the observed shortening was a specific result of ABCC4 loss (Fig. S1 D′). To probe the effect of ABCC4 loss on SHH signaling, we analyzed SMO ciliary accumulation as an indicator of signal initiation and GLI transcriptional activity as a read-out of downstream signal transduction. Intriguingly, SHH-stimulated SMO ciliary entry was maintained in *Abcc4*^*−/−*^ cells, indicating that initiation of the SHH signal response can occur in cells lacking ABCC4 function (Fig. 1 B). Despite this, SMO communication with GLI transcriptional effectors was compromised (Fig. 1 B′), which is consistent with defective downstream transduction of the SHH activation signal. Based on these results, we concluded that ABCC4 transporter function contributes to ciliary length maintenance and SHH signal transduction downstream of SMO.

Our previous work demonstrated that activation of cPLA_2_α promotes SMO PC enrichment and signaling (Arensdorf et al., 2017). To determine whether cPLA_2_α also contributes to ciliary length maintenance, we tested the effect of cPLA_2_α inhibition on IMCD3 PC length. IMCD3 cells were treated with vehicle or GIRI in the absence and presence of SHH, and lengths of acetylated α-tubulin-marked PC were measured. Inhibition of cPLA_2_α shortened PC significantly in both basal and SHH-stimulated conditions. The average PC length was ∼5 μm in the absence of GIRI and was reduced by ∼40% to ∼3 μm at the highest drug concentration used (Fig. 1, C and C′). PC length of GIRI-treated cells was rescued by the addition of supplemental PGE_2_ or its precursor arachidonic acid, supporting the specificity of GIRI effects and suggesting that cPLA_2_α promotes ciliary length control through arachidonic acid production (Fig. 1, D and D′; and Fig. S1, E and E′, columns 5–7). Notably, supplemental arachidonic acid augmented ciliary length in SHH-stimulated cells, further supporting the hypothesis that there is a correlation between increased arachidonic acid availability and PC elongation (Fig. S1 E′, column 2 versus 3–4). Taken together, these results suggest that the PC shortening observed in GIRI-treated cells was a specific effect of compromised arachidonic acid-to-PGE_2_ metabolism.

To determine whether cPLA_2_α inhibition impacted SMO ciliary accumulation in IMCD3 cells, we stimulated cells with SHH-conditioned media in the absence or presence of GIRI and then quantified SMO ciliary signal intensity. Consistent with what we observed in NIH-3T3 cells (Arensdorf et al., 2017), cPLA_2_α inhibition reduced SMO ciliary occupancy in SHH-treated IMCD3 cells (Fig. S1, F and F′, red). SHH-stimulated ciliary exit of GPR161 (magenta) was unaffected by GIRI treatment (Fig. S1, F and F′), suggesting that the effects of cPLA_2_α inhibition may be specific to SHH-promoted anterograde ciliary transport of SMO. Combined with the above, these results suggest that cPLA_2_α-generated arachidonic acid contributes to SHH signaling in two manners: (1) it drives a feed-forward signal that promotes SMO ciliary accumulation (Arensdorf et al., 2017), and (2) it facilitates the production of PGE_2_, which is secreted by ABCC4 to maintain ciliary length for robust SMO signaling to GLI.

### EP_4_ activation promotes ciliary length homeostasis for SHH signaling to GLI

**Figure 2.**
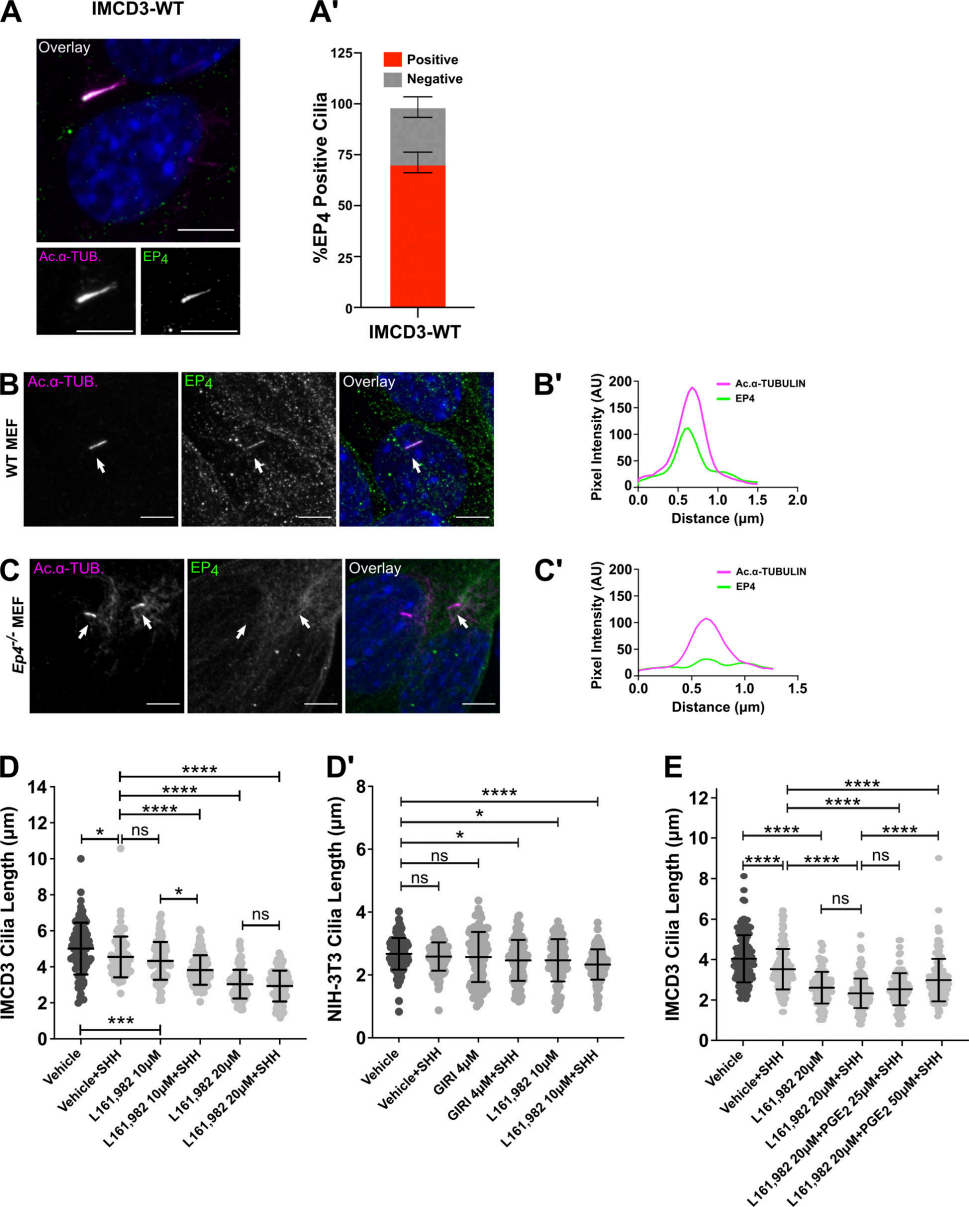
**EP**_**4**_ **is required for PC length homeostasis. (A)** Endogenous EP_4_ is shown in green and acetylated α-tubulin (magenta) marks the PC in an IMCD3 cell. Single channels are shown in grayscale. Scale bar = 5 µm. **(A′)** EP_4_ localization was scored in IMCD3 cells. Approximately, 75 cells were analyzed across two experiments and all data were pooled. **(B–C′)** EP_4_ localizes to PC in wild-type MEFs and the signal is lost in *Ep4*^*−/−*^ cells. Line scans indicate the degree of localization between EP_4_ (green) and acetylated α-tubulin (magenta) in (B′) control and (C′) *Ep4*^*−/−*^ MEFs. Intensity profiles are presented as arbitrary units (AU). Scale bars = 5 μm. **(D and D′)** EP_4_ inhibition shortens PC in IMCD3 (D) and NIH-3T3 (D′) cells. Cells were pretreated with vehicle (DMSO) or EP_4_ inhibitor L161,982 (10 or 20 µM) for 2 h prior to the addition of SHH or control conditioned media. **(E)** IMCD3 cells were treated with control or SHH-conditioned media in the presence of vehicle or 20 µM L161,982 in media supplemented with increasing concentrations of PGE_2_ (25 or 50 μM). At least 150 cilia/condition were measured over three experiments. Statistical significance was calculated using one-way ANOVA. For all experiments, a P value of <0.05 was considered statistically significant with significance indicated as follows: *<0.05, **<0.01, ***<0.001, ****<0.0001, and ns, P > 0.05. Data are represented as mean ± SD.

**Figure S2.**
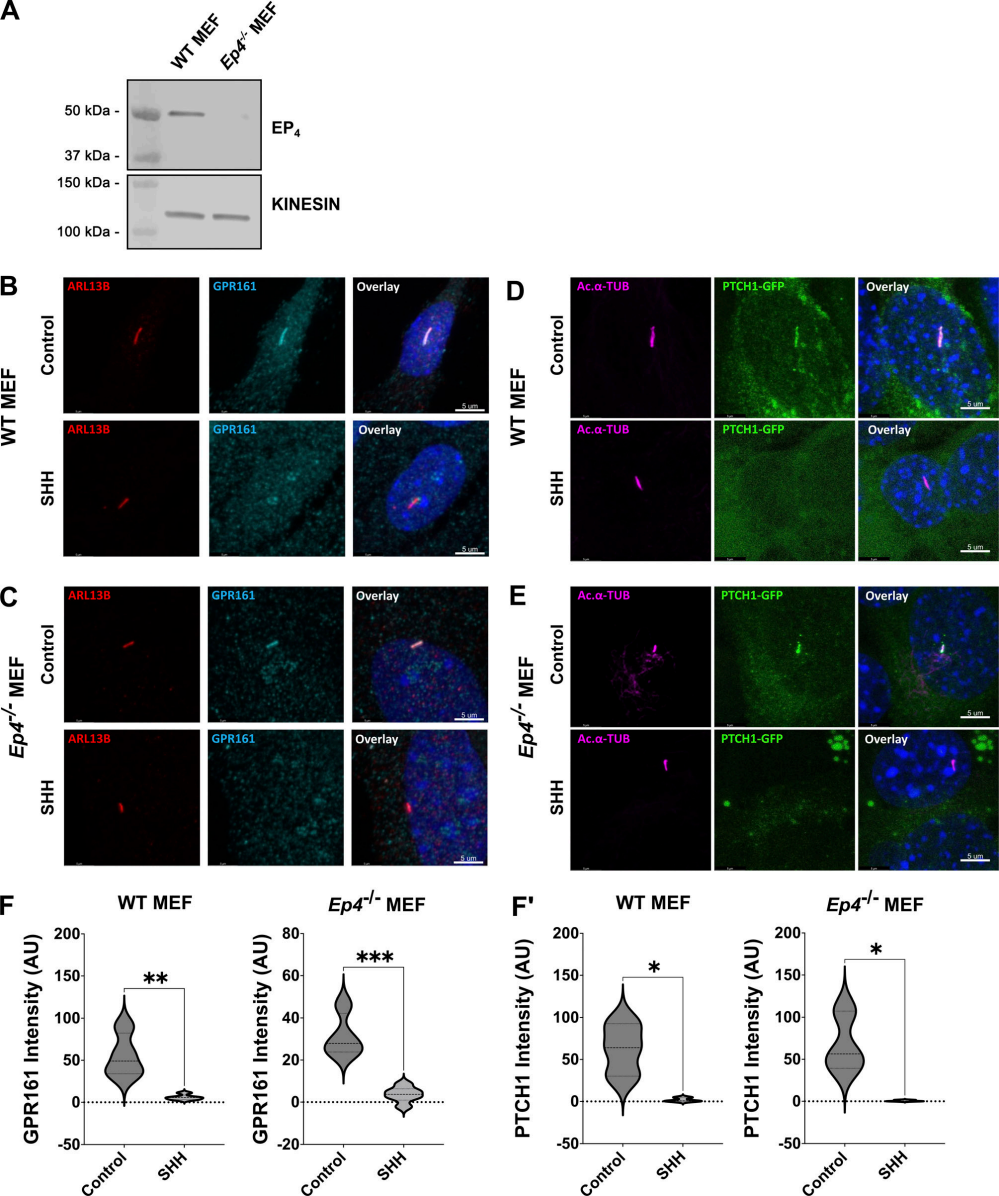
**Analysis of of *Ep4^−/−^* MEFs. (A)** Western blot showing expression level of EP_4_ in control and *Ep4*^*−/−*^ MEFs. Kinesin is the loading control. **(B and C)** Confocal images of GPR161 in control and *Ep4*^*−/−*^ MEFs are shown. GPR161 is in cyan, ARL13B (red) marks primary cilia. Scale bar = 5 μm. **(D and E)** Confocal images of PTCH1-GFP are shown in control and *Ep4*^*−/−*^ MEFs following SHH stimulation. PTCH1-GFP is green and acetylated α-tubulin (magenta) marks PC. Scale bar = 5 μm. **(F and F′)** Quantification of ciliary signal intensity for (F) GPR161 and (F′) PTCH1. The experiment was performed twice with 20 cilia imaged per condition per experiment. Significance was determined using a Student’s *t* test. Significance is indicated as follows: *<0.05, **<0.01, ***<0.001. Source data are available for this figure: SourceData FS2.

Based on established connections between EP_4_ and motile cilia elongation (Jin et al., 2014; Marra et al., 2019), we hypothesized that PGE_2_ produced downstream of SHH activates ciliary EP_4_ to influence PC length. Consistent with it being a ciliary GPCR, EP_4_ was observed in PC of both IMCD3 cells and control mouse embryonic fibroblasts (MEF) and was not detected in cilia of *Ep*_*4*_^*−/−*^ MEFs (Fig. 2, A–C′; and Fig. S2 A). To test the contribution of EP_4_ to PC length maintenance, we compared the average ciliary lengths of control and SHH-treated IMCD3 cells exposed to vehicle or the EP_4_ inhibitor, L161,982 (Cherukuri et al., 2007). Cilia of IMCD3 cells treated with increasing concentrations of L161,982 were shortened in a dose-dependent manner in the absence of SHH, and shortening was increased by SHH treatment (Fig. 2 D). Statistically significant ciliary shortening was also observed in SHH-stimulated NIH-3T3 cells treated with GIRI or L161,982 (Fig. 2 D′). This suggests that SHH stimulation compromises PC length maintenance if EP_4_ activity is reduced. Importantly, supplementing the culture media of L161,982-treated IMCD3 cells with the EP_4_ ligand PGE_2_ partially rescued the ciliary length of SHH-stimulated cells (Fig. 2 E). This suggests that PC shortening following L161,982 treatment was a specific effect of on-target inhibitor activity toward EP_4_.

**Figure 3.**
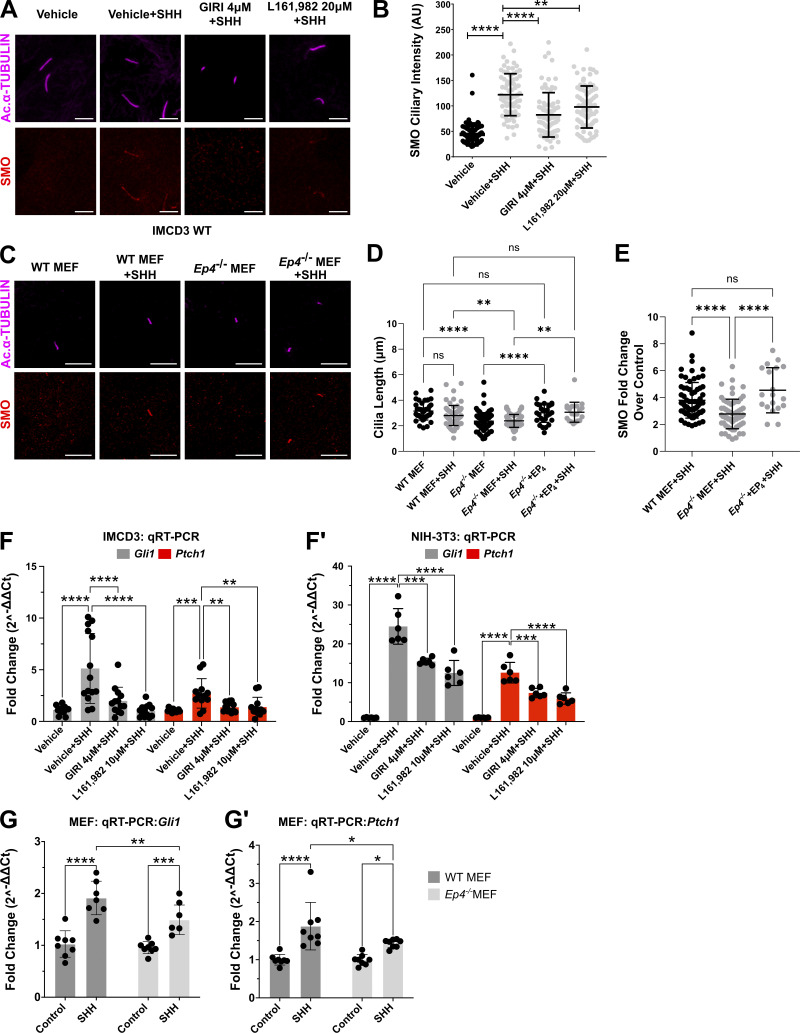
**SHH signaling is attenuated by inhibition of cPLA**_**2**_**α or EP**_**4**_**. (A)** SMO (red) enrichment in acetylated α-tubulin (magenta) marked PC was examined in IMCD3 cells. Cells were pretreated with vehicle (DMSO), GIRI (4 µM), or L161,982 (20 µM) for 18 h. Scale bar = 5 μm. **(B)** Average SMO ciliary signal intensity was quantified for each condition. Approximately, 50 cilia were analyzed across two experiments, and all data were pooled. **(C–E)** SMO (red) PC enrichment and ciliary length were examined in wild type and *Ep4*^*−/−*^ MEFs minus or plus EP_4_ re-expression 18 h after treatment with control or SHH-conditioned media. **(C)** Cilia are marked by acetylated α-tubulin (magenta). Scale bar = 10 μm. Quantification of (D) average PC length and (E) fold change in SMO signal intensity are shown. Approximately, 50 cilia across two experiments were analyzed and all data were pooled. **(F and F′)** qRT-PCR analyses of *Gli1* and *Ptch1* expression in (F) IMCD3 and (F′) NIH-3T3 cells were performed. Cells were pretreated with vehicle (DMSO), GIRI (4 µM), or L161,982 (10 µM) for 2 h and then cultured in control or SHH-conditioned media plus inhibitor for 18 h. **(G and G′)** qRT-PCR analyses of *Gli1* and *Ptch1* expression in control and *Ep4*^*−/−*^ MEF cells were performed. Fold change in expression was determined using the 2^–∆∆Ct^ method. Average fold change was calculated across at least two independent experiments with three biological replicates per experiment. All data are pooled. For all experiments, statistical significance was determined using a one-way ANOVA. A P value of <0.05 was considered statistically significant. Significance is denoted as follows: *<0.05, **<0.01, ***<0.001, ****<0.0001, and ns, P > 0.05. Error bars indicate SD.

To understand how EP_4_ inhibition affected SHH signal output, SHH-stimulated SMO ciliary signal intensity was quantified in control, GIRI-treated, and L161,982-treated IMCD3 cells. Similar to what occurred following cPLA_2_α inhibition with GIRI, inhibition of EP_4_ with L161,982 attenuated accumulation of endogenous SMO in PC following SHH exposure (Fig. 3, A and B). Ciliary shortening and reduced SMO ciliary accumulation relative to control were also observed in *Ep4*^*−/−*^ MEFs (Fig. 3, C–E). Both PC length and SMO ciliary accumulation were restored by the reintroduction of EP_4_ into *Ep4*^*−/−*^ MEFs (Fig. 3, D and E), supporting the hypothesis that the effects of L161,982 treatment on PC length and SMO ciliary accumulation resulted from on-target inhibition of EP_4_. To determine whether reduced SMO ciliary enrichment following EP_4_ inhibition compromised the activation of GLI transcriptional effectors, qPCR analyses of SHH transcriptional targets *Gli1* and *Ptch1* were performed in IMCD3 cells, NIH-3T3 cells, and *Ep4*^*−/−*^ MEFs. Inhibition of either cPLA_2_α with GIRI or EP_4_ with L161,982 significantly reduced the ability of SHH to activate a transcriptional response in IMCD3 and NIH-3T3 cells (Fig. 3, F and F′). SHH-stimulated target gene induction was similarly reduced in *Ep4*^*−/−*^ MEFs, despite the ability of PTCH1 and GPR161 to exit cilia of cells lacking EP_4_ function (Fig. 3, G and G′; and Fig. S2, B–F′). Thus, compromised PGE_2_/EP_4_ signaling shortens cilia and attenuates downstream SHH pathway activity.

### EP_4_ signaling promotes ciliary cAMP recovery following SHH pathway activation

**Figure 4.**
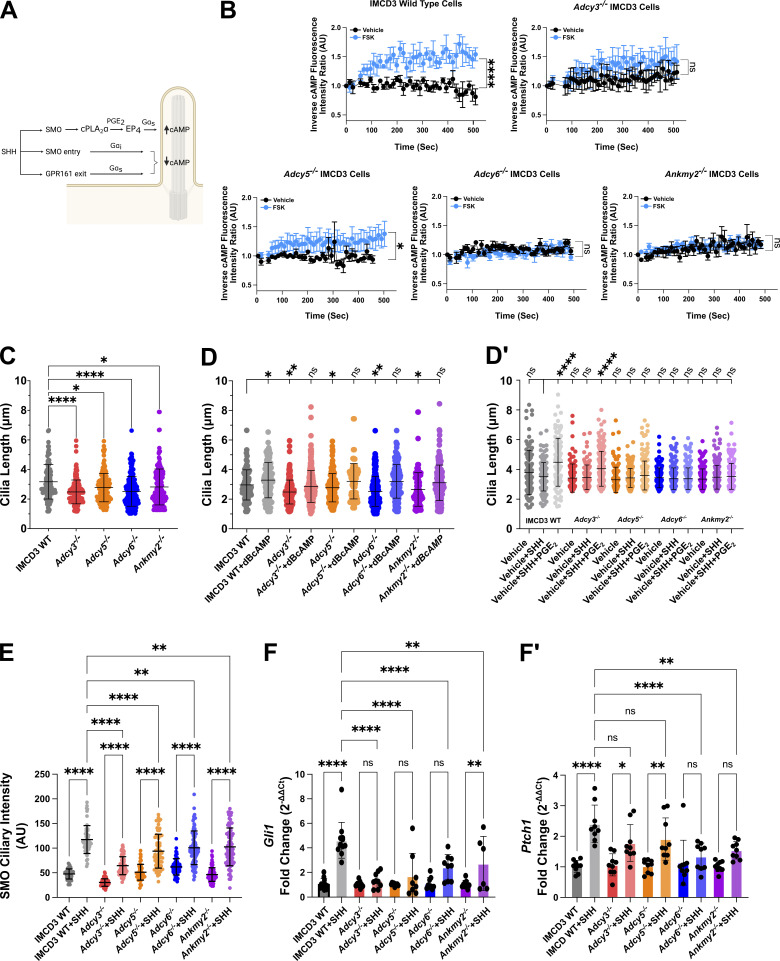
**Ciliary adenylyl cyclases (AC) contribute to PC length control and SHH-stimulated transcriptional activation. (A)** A model for SHH and EP_4_ effects on PC cAMP. **(B)** Ciliary cAMP was measured in control, *Adcy3*^*−/−*^, *Adcy5*^*−/−*^, *Adcy6*^*−/−*^, and *Ankmy2*^*−/−*^ IMCD3 cells using the cADDis cAMP sensor. cADDis activity was recorded as cells were exposed to the vehicle (ethanol, black) or the AC activator forskolin (FSK, 100 μM, blue). Ciliary fluorescence intensity was recorded over 8 min in live cell imaging mode. An average of ∼5 cilia were recorded for each condition. The experiment was performed twice. A representative experiment is shown. Significance was determined by calculating the area under the curve followed by Student’s *t*-Test analysis. **(C)** Average ciliary lengths are shown for control, *Adcy*^*−/−*^, and *Ankmy2*^*−/−*^ IMCD3 cells. Approximately 100 cilia across three experiments were measured and all data were pooled. **(D and D′)** Average ciliary lengths are shown for control, *Adcy*^*−/−*^, and *Ankmy2*^*−/−*^ IMCD3 cells treated with (D) dBcAMP (100 μM) and (D′) SHH or SHH + PGE_2_ (40 μM). Cells were pretreated with vehicle (DMSO) for 2 h prior to the addition of dBcAMP, SHH, or SHH + PGE_2_. PC length was measured 18 h after stimulation for at least 150 cilia/condition over two independent experiments. Statistical significance was calculated using one-way ANOVA. All ciliary lengths were compared to vehicle-treated control IMCD3 cells in D and vehicle + SHH-treated IMCD3 cells in D′. **(E)** SMO ciliary signal intensity was determined in control, *Adcy*^*−/−*^, and *Ankmy2*^*−/−*^ IMCD3 cells treated with control or SHH-conditioned media. Approximately 75 cilia across two experiments were analyzed and all data were pooled. **(F and F′)** qRT-PCR analyses of *Gli1* and *Ptch1* expression in control, *Adcy*^*−/−*^, and *Ankmy2*^*−/−*^ IMCD3 cells were performed. Fold change in expression was determined using the 2^–∆∆Ct^ method. The average fold change was calculated across three independent experiments. Fold change over control conditioned media treatment was calculated using the 2^–∆∆Ct^ method. Significance was determined by one-way ANOVA. For all experiments, a P value of <0.05 was considered statistically significant. Significance is denoted as follows: *<0.05, **<0.01, ***<0.001, ****<0.0001, and ns, P > 0.05. Error bars indicate SD.

**Figure S3.**
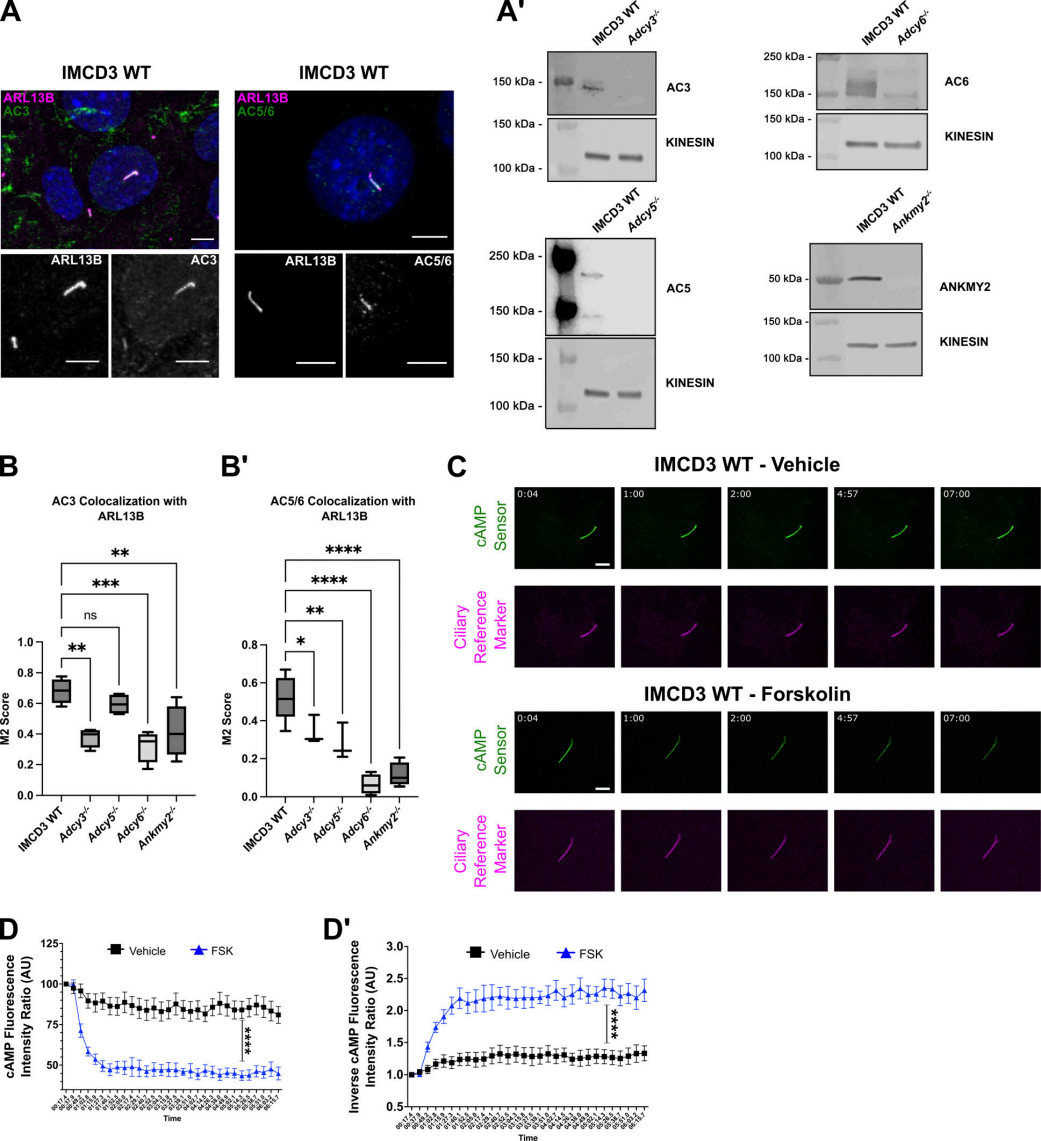
**Examination of ciliary AC localization and cAMP dynamics. (A)** AC3 and AC5/6 localize to primary cilia in wild-type IMCD3 cells. ARL13B (magenta in merge and white in insets) marks primary cilia. Ciliary ACs are shown in green (merged) and in white (insets). Scale bar = 5 μm. **(A′)** Western blots for AC3, AC5, AC6, and ANKMY2 in lysates from control or pooled knockout IMCD3 cells. Kinesin is the loading control. **(B and B′)** Colocalization (M2 score) of (B) AC3 and (B′) AC5/6 with ARL13B in primary cilia of control, *Adcy3*^*−/−*^, *Adcy5*^*−/−*^, *Adcy6*^*−/−*^, and *Ankmy2*^−/−^ cells. Statistical significance was determined using a one-way ANOVA. **(C–D′)** Time points from imaging of cADDis-expressing IMCD3 cells show changes in fluorescence intensity of a ciliary cAMP sensor (green) and stable fluorescence of the ciliary reference marker (magenta) following exposure to vehicle or forskolin (FSK, 100 µM). Increasing cAMP decreases green fluorescence. Scale bar = 2 μm. **(D and D′)** The relative cAMP shift is shown as the native (D) or (D′) inverse of the fluorescent intensity ratio of the cAMP sensor to reference marker. Inverse ratios are shown in the main figures to clearly illustrate cAMP increase. Significance was determined by calculating the area under the curve followed by Student’s *t* Test analysis. Significance is indicated as follows: *<0.05, **<0.01, ***<0.001, ****<0.0001, and ns, P > 0.05. Source data are available for this figure: SourceData FS3.

We hypothesized that coordinated GPCR regulation of ciliary cAMP production facilitates PC length homeostasis in SHH-stimulated cells (Fig. 4 A). To interrogate this hypothesis, we compromised ciliary cAMP production by knocking out the ciliary ACs *Adcy3*,* Adcy5*, and *Adcy6* and the ciliary AC chaperone *Ankmy2* using CRISPR/Cas9 (Fig. S3, A–B′) (Somatilaka et al., 2020). AC or ANKMY2 protein reduction in knockout cell pools was validated by Western blot, and ciliary AC signal reduction was assessed by analyzing colocalization with the ciliary resident protein ARL13B (Fig. S3, A′–B′). Notably, knockout of individual ACs reduced ciliary localization of the remaining ACs, which could indicate shared trafficking mechanisms (Fig. S3, B and B′). We used a PC-localized fluorescent sensor system to track ciliary cAMP changes in knockout cell pools and observed that loss of each of the ciliary *Adcys* and *Ankmy2* blocked ciliary cAMP accumulation following IMCD3 treatment with the AC-stimulating drug forskolin (FSK, Fig. 4 B and Fig. S3, C–D′). Consistent with our hypothesis that reduced ciliary cAMP production leads to PC shortening, we observed statistically significant length decreases following knockout of *Adcy3*, *Adcy5*, *Adcy6*, or *Ankmy2* (Fig. 4 C). PC length was restored in each of the knockout cell pools by supplementing culture media with the cAMP analog dibutyryl-cAMP (db-cAMP, Fig. 4 D), suggesting that ciliary cAMP reduction led to PC shortening following ciliary AC depletion. Consistent with cAMP effects on ciliary length occurring through EP_4_ activation, control IMCD3 cells showed PC elongation following exposure to SHH and the EP_4_ agonist PGE_2_. PC elongation was also observed in *Adcy3*^*−/−*^ cells stimulated with PGE_2_, but similar length increases were not observed in *Adcy5*^*−/−*^, *Adcy6*^*−/−*^, or *Ankmy2*^*−/−*^ cells, suggesting that cAMP produced downstream of SHH and PGE_2_ occurs primarily through AC5 and AC6 (Fig. 4 D′, gray and pink versus yellow and blue). Taken together, these results suggest that SHH-promoted EP_4_ signaling activates ciliary ACs to raise ciliary cAMP for PC length homeostasis.

**Figure S4.**
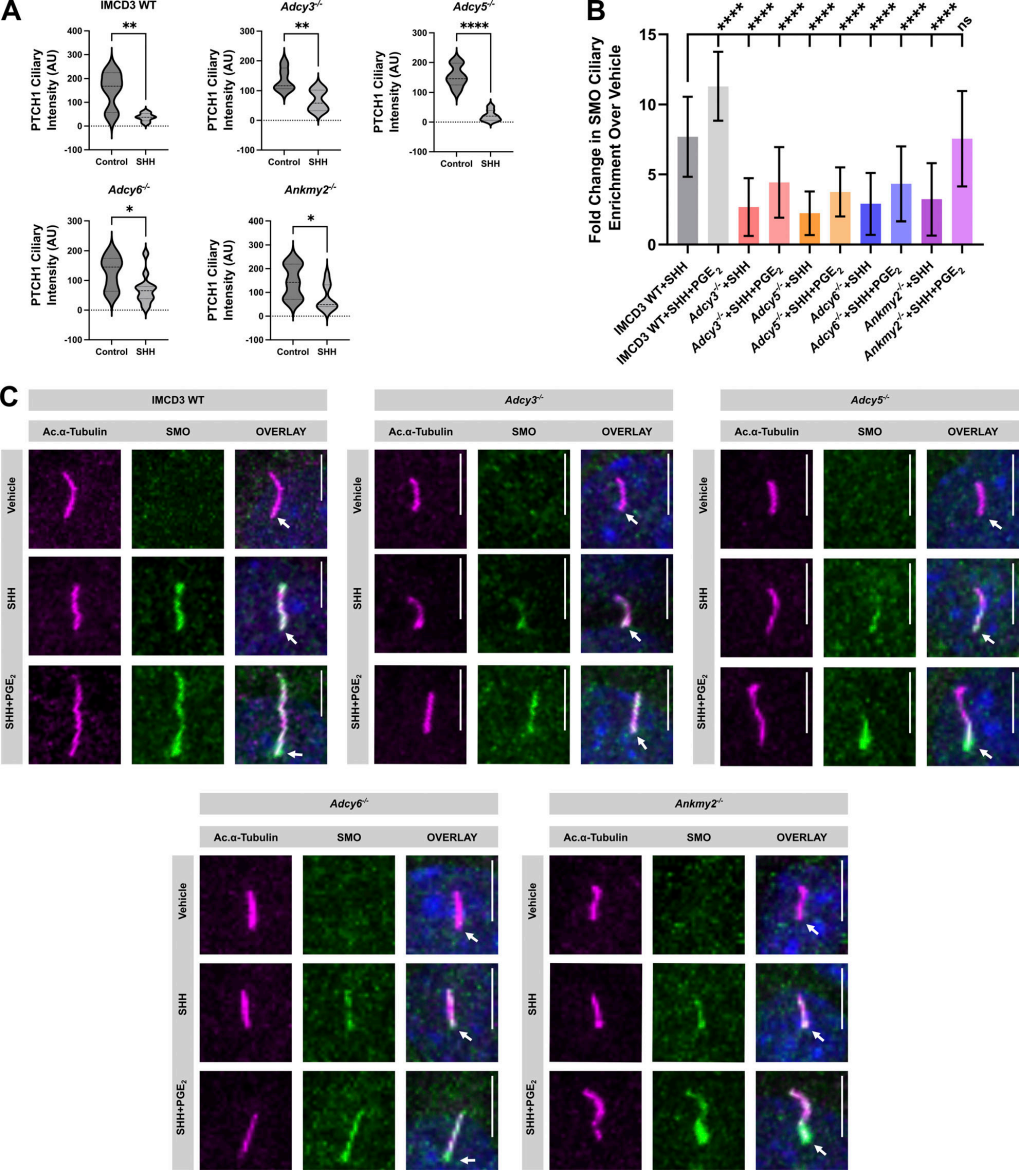
**Evaluation of PTCH1 and SMO ciliary localization in control and AC knockout cells. (A)** Quantification of PTCH1-GFP ciliary signal intensity is shown for control, *Adcy3*^*−/−*^, *Adcy5*^*−/−*^, *Adcy6*^*−/−*^, and *Ankmy2*^−/−^ IMCD3 cells. 24 h after transfection, cells were serum starved for 2 h prior to the addition of either control or SHH-conditioned media. PTCH1 ciliary intensity was measured after 18 h of stimulation. **(B)** SMO ciliary intensity was measured in control, *Adcy*^*−/−*^, and *Ankmy2*^*−/−*^ IMCD3 cells following vehicle, SHH, or SHH + PGE_2_ (40 μM) treatment. Cells were pretreated with vehicle (DMSO) for 2 h prior to the addition of SHH or SHH + PGE_2_. SMO intensity was measured after 18 h of stimulation. SMO intensity fold change over vehicle control is shown. **(C)** Immunofluorescence of PC in control, *Adcy3*^*−/−*^, *Adcy5*^*−/−*^, *Adcy6*^*−/−*^, and *Ankmy2*^−/−^ IMCD3 cells is shown following treatment with vehicle, SHH, or SHH + PGE_2_. The ciliary axoneme is marked by ARL13B (magenta), SMO is green and DAPI (blue) marks the nucleus. Arrowheads mark the PC base. Scale bar = 5 μm. Significance was determined by A Student’s *t* Test or B one-way ANOVA. For all experiments, a P value of <0.05 was considered statistically significant. Significance is denoted as follows: *<0.05, **<0.01, ***<0.001, ****<0.0001, and ns, P > 0.05. Data are represented as mean intensities ± SD.

To determine whether PC shortening in knockout cells correlated with reduced SHH pathway activation and downstream signaling, we compared SMO ciliary enrichment and target gene induction between control and knockout cell pools. Whereas SHH promoted PTCH1 exit and SMO ciliary accumulation in each of the knockout cell pools, the level of SMO ciliary enrichment was reduced compared with control cells (Fig. 4 E and Fig. S4 A). Notably, SMO was distributed along the PC membrane and was evident at PC tips in control cells exposed to either SHH or SHH + PGE_2_, but did not efficiently spread along PC membranes in knockout cell pools in response to these ligands (Fig. S4, B and C). Curiously, the SMO ciliary enrichment that was observed in knockout cell pools treated with SHH or SHH + PGE_2_ occurred near the PC base. Base accumulation was particularly pronounced in *Ankmy2*^*−/−*^ cells exposed to SHH + PGE_2_, suggesting that pan-depletion of ciliary AC activity significantly stalls anterograde SMO ciliary trafficking (Fig. S4 C, arrows). Consistent with high-level SMO signaling requiring its accumulation throughout the PC, SHH-stimulated *Gli1* transcriptional activation was reduced in knockout cells (Fig. 4 F). SHH-stimulated *Ptch1* induction was also compromised, albeit to a lesser extent (Fig. 4 F′). Thus, we conclude that the ability to produce cAMP in the PC is necessary to stabilize ciliary length and promote SMO PC occupancy. When this capability is lost, SHH target gene activation is compromised.

**Figure S5.**
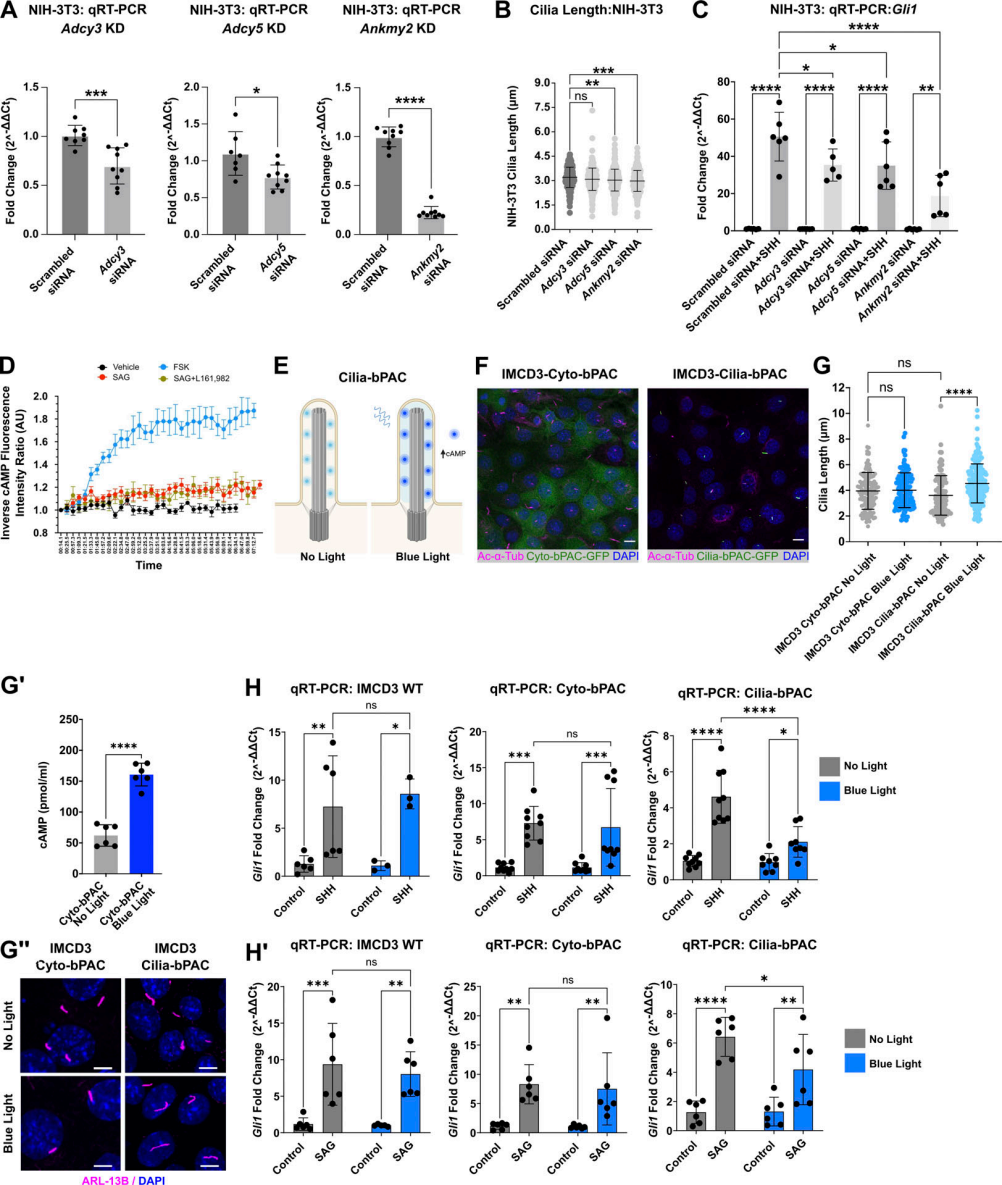
**Evaluation of primary cilium length and SHH transcriptonal output following ciliary cAMP modulation. (A)** *Adcy3*, *Adcy5*, and *Anmky2* knockdown in NIH-3T3 cells was validated by qRT-PCR ∼48 h after treatment with *Adcy3*, *Adcy5*, or *Ankmy2* siRNA or scrambled control. Fold-change in expression was determined using the 2^–∆∆Ct^ method. Significance was determined using Student’s *t* Test. Significance is indicated as follows: *<0.05, **<0.01, ***<0.001, ****<0.0001, and ns, P > 0.05. **(B)** Cilia were measured for control, *Adcy3*, *Adcy5*, or *Ankmy2* NIH-3T3 cells. Approximately 150 cilia were measured across two experiments, and all data were pooled. **(C)** *Gli1* fold change was determined in control or SHH-stimulated NIH-3T3 cells following *Adcy3*, *Adcy5*, or *Ankmy2* knockdown. Knockdown experiments were repeated 3 times with ∼75 primary cilia measured per experiment. Significance was determined by one-way ANOVA. **(D)** cADDis-expressing IMCD3 cells were pretreated overnight with vehicle or L161,982 (10 µM). The following morning, cADDis activity was monitored by live imaging to track the ciliary cAMP response as cells were treated with or without forskolin (FSK, 100 μM) for 1.5 min prior to addition of SAG (1 μM) or vehicle control. Ciliary fluorescence intensity was recorded over 8 min in live cell imaging mode. An average of ∼six cilia were recorded for each condition and the experiment was performed twice. A representative experiment is shown. **(E)** Schematic of optogenetic cAMP modulation in cilia. **(F)** Immunofluorescence imaging of IMCD3 cells expressing Cyto-bPAC-GFP or Cilia-bPAC-GFP (green) showed expression in the expected cell compartments. Acetylated α-tubulin marks cilia (magenta) and DAPI (blue) marks nuclei. Scale bars = 10 μm. **(G)** Average PC length was quantified in Cyto-bPAC and Cilia-bPAC expressing IMCD3 cells in the dark or following 3 h of blue light exposure. **(G′)** Total cellular cAMP was measured in Cyto-bPAC IMCD3 cells between resting and blue light stimulated conditions. Error bars indicate SD. Significance was determined by (G) one-way ANOVA and (G′) Student’s *t* test. **(G″)** PC of Cyto-bPAC or Cilia-bPAC expressing IMCD3 cells were imaged by confocal microscopy following 3 h of blue light exposure. Acetylated α-tubulin is magenta and DAPI is blue. Scale bar = 5 μm. **(H and H′)** qRT-PCR measurement of *Gli1* expression in control, Cyto-bPAC or Cilia-bPAC expressing IMCD3 cells. Cells were stimulated overnight after 2 h of starvation with either (H) SHH conditioned media or (H′) SAG (100 nM) followed by either dark incubation or blue light treatment. Fold change over vehicle control was calculated using the 2^–∆∆Ct^ method replicates. The experiment was repeated at least twice with three biological replicates per experiment. All data were pooled. Significance was assessed using one-way ANOVA. For all experiments, significance is denoted as follows: *<0.05, **<0.01, ***<0.001, ****<0.0001, and ns, P > 0.05. Data are represented as mean ± SD.

Our observations that decreased IMCD3 ciliary AC occupancy correlated with reduced target gene induction were curious because ANKMY2 loss in vivo leads to SHH-independent GLI stabilization and increased transcriptional activity in the developing neural tube (Somatilaka et al., 2020). Thus, we expanded our evaluation to include highly SHH-responsive NIH-3T3 cells. We did not detect endogenously expressed *Adcy6* transcript in NIH-3T3 cells, so we did not evaluate its knockdown in this cell type. Due to baseline PC lengths in NIH-3T3 cells being shorter than those of IMCD3 cells, length reductions occurring in response to ciliary AC depletion appeared modest. Nevertheless, we observed that knockdown of *Adcy5* or *Ankmy2* led to statistically significant reductions in average PC lengths in NIH-3T3 cells (Fig. S5, A and B). *Adcy3* knockdown did not significantly alter NIH-3T3 ciliary length, suggesting that AC5 may be the primary modulator of cAMP-mediated PC length regulation in this cell type (Fig. S5 B). However, we did observe a modest reduction in SHH-stimulated *Gli1* transcriptional activation following knockdown of either *Adcy3* or *Adcy5*, suggesting that ciliary localization of both ACs may be necessary to optimize PC cAMP control for downstream GLI regulation in SHH-stimulated cells. Consistent with this hypothesis, *Ankmy2* knockdown, which is predicted to cause pan-AC ciliary depletion, triggered a more pronounced reduction in SHH-stimulated *Gli1* induction than that observed following a single AC knockdown (Fig. S5 C). These observations provide additional evidence that PC cAMP depletion can reduce PC length and blunt ligand-induced SHH signal output.

**Figure 5.**
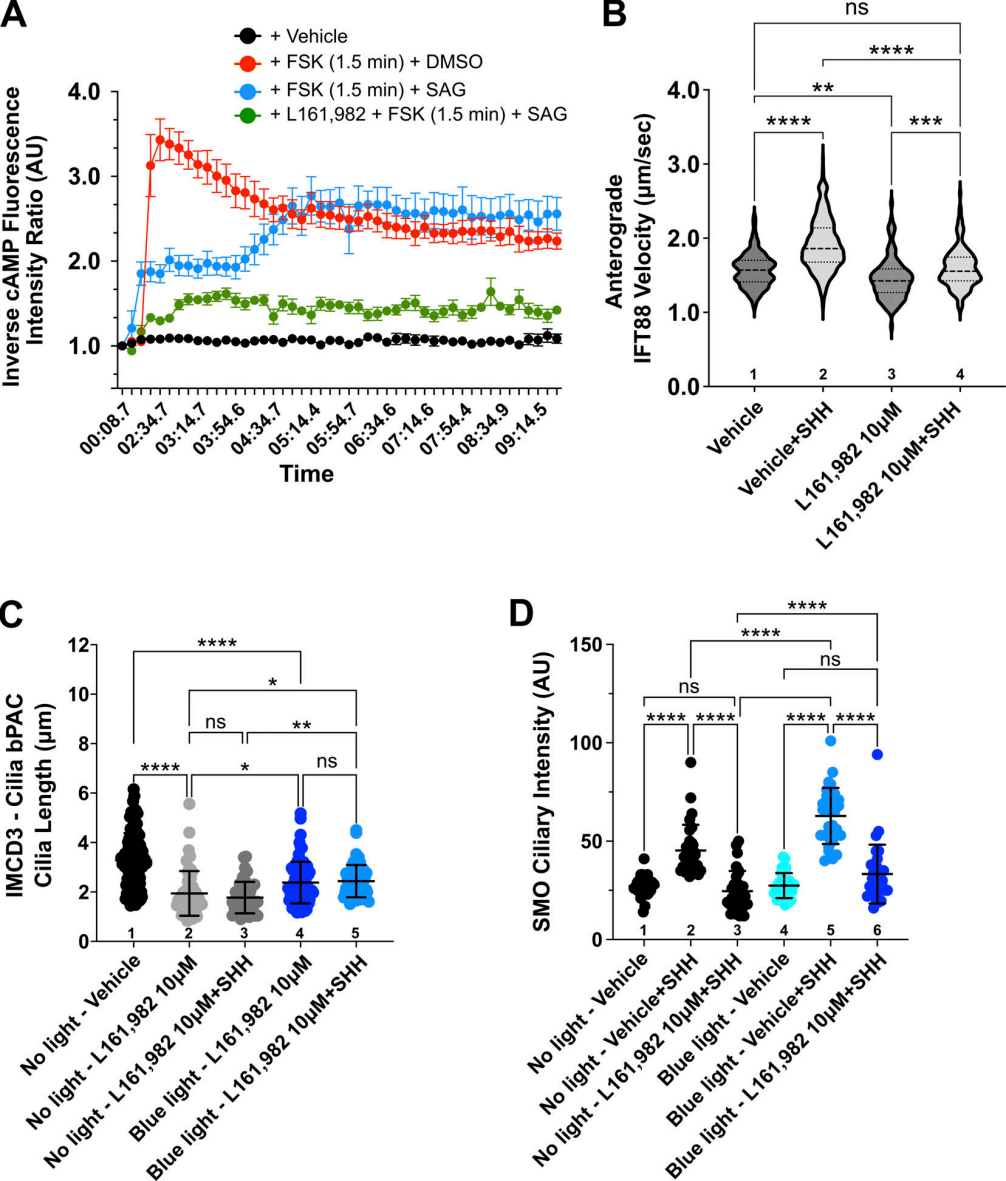
**EP**_**4**_ **stabilizes ciliary cAMP in SHH-stimulated cells to maintain anterograde IFT and promote SMO ciliary accumulation. (A)** cADDis-expressing IMCD3 cells were pretreated overnight with vehicle or L161,982 (10 µM). The following morning, cADDis activity was monitored by live imaging to track the ciliary cAMP response as cells were treated with forskolin (FSK, 100 μM) for 1.5 min prior to the addition of the SMO agonist SAG (1 μM) or vehicle control. Fluorescence ciliary intensity was recorded over 10 min in live cell imaging mode. An average of ∼six cilia were recorded for each condition and the experiment was performed twice. A representative experiment is shown. **(B)** Anterograde IFT velocity was calculated in IMCD3 cells by tracking IFT88-GFP movement in the presence and absence of SHH, L161,982 (10 µM), or vehicle control. IFT velocity was calculated for 30 cilia per condition across four experiments. Velocity is shown as a violin plot with SD indicated. **(C and D)** Average ciliary length and SMO ciliary intensity were quantified in IMCD3-bPAC cells exposed to control or SHH-conditioned media in the absence or presence of 10 µM L161,982 in control or blue light-exposed cells. Significance was determined by one-way ANOVA. For all experiments, a P value of <0.05 was considered statistically significant. Significance is denoted as follows: *<0.05, **<0.01, ***<0.001, ****<0.0001, and ns, P > 0.05. Data are represented as mean ± SD. For all ciliary length and SMO ciliary intensity experiments, 50–100 cells per condition were analyzed over at least three independent experiments.

Having found that lowering ciliary cAMP via AC depletion shortens PC and attenuates SHH activity in IMCD3 and NIH-3T3 cells, we next tested whether SMO-regulated cAMP dynamics were influenced by EP_4_. Because baseline ciliary cAMP levels were too low for the fluorescent ciliary cAMP reporter to detect cAMP reduction following SMO activation or EP_4_ inhibition (Fig. S5 D), IMCD3 cells were stimulated with the AC activator FSK to raise basal cAMP prior to SMO stimulation (Fig. 5 A, red). Costimulation of IMCD3 cells with FSK and the direct SMO agonist SAG reduced the level to which FSK could raise ciliary cAMP, which is consistent with the ability of active SMO to couple with AC inhibitory Gα_i_. Despite this, ciliary cAMP in cells treated with FSK + vehicle and FSK + SAG equilibrated similarly after an initial lag (Fig. 5 A, red versus blue). We hypothesized cAMP equilibration in SAG-treated cells was the result of EP_4_ activation downstream of SMO-stimulated PGE_2_ release. Accordingly, ciliary cAMP equilibration in SAG-stimulated cells was blocked by direct EP_4_ inhibition with L161,982 (Fig. 5 A, green versus blue). Thus, we posit that EP_4_ activation downstream of SMO contributes to the equilibration of PC cAMP levels in SHH-stimulated cells.

Previously published studies have indicated that EP_4_ raises ciliary cAMP to promote anterograde IFT (Besschetnova et al., 2010; Jin et al., 2014). To determine if IFT was compromised by EP_4_ inhibition in SHH-stimulated cells, we calculated the anterograde velocity of the ciliary transport protein IFT88 in control and L161,982-treated cells. We observed increased anterograde IFT in vehicle-treated, SHH-stimulated cells (Fig. 5 B, lane 1 versus 2). Anterograde IFT88 velocity was significantly reduced following treatment with the EP_4_ inhibitor L161,982 in both control and SHH-stimulated IMCD3 cells (Fig. 5 B, lane 3 versus 4). These results suggest that EP_4_ signaling maintains anterograde IFT in control cells and enhances its velocity in SHH-stimulated cells.

If the SHH-regulated cAMP equilibration that ensures IFT maintains PC length occurs through SHH-to-EP_4_ crosstalk, we reasoned that supplemental ciliary cAMP should restore ciliary length and rescue SHH pathway induction following EP_4_ inhibition. To modulate ciliary cAMP in a controlled manner, we used a published optogenetic AC system (Truong et al., 2021). IMCD3 cells were engineered to stably express either cytoplasmic or PC-localized bacterial photoactivatable adenylyl cyclase (bPAC)-GFP (Fig. S5, E and F). IMCD3 cells expressing cytoplasmic or ciliary bPAC proteins showed similar average PC lengths in the absence of blue light stimulation. PC lengths of cyto-bPAC-expressing cells did not change upon blue light exposure, despite increased cytoplasmic cAMP levels (Fig. S5, G–G″). Conversely, cilia-bPAC-expressing cells increased their average PC length by ∼30% upon blue light exposure (Fig. S5, G and G″), which is consistent with published evidence that PC-specific stimulation of cAMP production can enhance ciliary length (Hansen et al., 2020).

Having validated the feasibility of using ciliary bPAC to modulate IMCD3 cell PC length, we next investigated whether shortened cilia of EP_4_-inhibited cells would elongate in response to cilia-bPAC induction. We observed that cilia-bPAC activation in L161,982-treated cells increased average PC length by 25% (Fig. 5 C, columns 2–3 versus 4–5). To test whether an increase in ciliary length correlated with the rescue of SHH pathway activity, we quantified SMO ciliary enrichment and GLI-mediated transcriptional activation following cilia-bPAC activation in the presence and absence of L161,982. Notably, SHH-stimulated SMO ciliary enrichment was significantly enhanced in vehicle-treated cells and partially restored in L161,982-treated cells in response to cilia-bPAC induction (Fig. 5 D, column 2 versus 5 and 3 versus 6). Despite this, the ability of SMO to induce GLI transcriptional activators following SHH or SAG treatment was blunted by cilia-bPAC stimulation (Fig. S5, H and H′). Similar to what has been previously reported, cyto-bPAC induction did not significantly alter the transcriptional response (Truong et al., 2021). These results, which are consistent with the established role of high ciliary cAMP promoting GLI repressor formation (Kong et al., 2019; Umberger and Ogden, 2021), suggest that an optimal balance of PC cAMP control must be achieved to maintain ciliary length homeostasis and anterograde SMO ciliary trafficking while allowing for downstream GLI activation.

### EP_4_ is required for ciliary length control and SHH signaling during neural tube development

**Figure 6.**
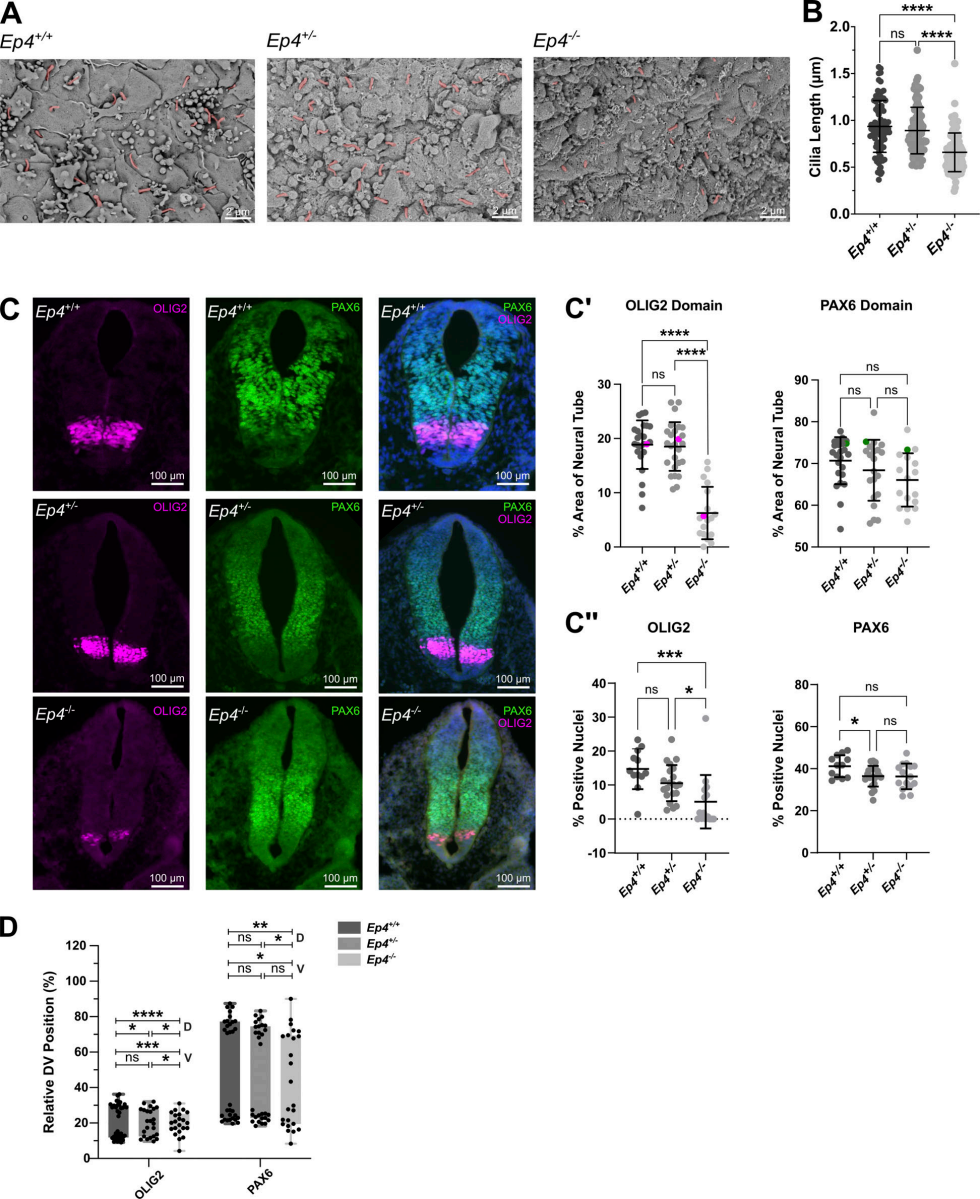
**EP**_**4**_ **loss reduces PC length and alters SHH-dependent neural tube patterning in vivo. (A)** Scanning electron micrographs of cardiac-level neural tube sections from E9.5 embryos of the indicated genotypes show apical membranes of cells lining the neural tube lumen. Scale bar = 2 μm. Images shown were taken from 27 somite-stage embryos. Cilia are pseudocolored in magenta. **(B)** The average PC length was calculated by measuring ∼30 cilia/section of cells lining the neural tube lumen. At least four/sections per embryo were analyzed across multiple embryos. **(C)** Cardiac level sections of E9.5 neural tubes from the indicated genotypes were immunostained for neural progenitor domain markers OLIG2 (magenta) and PAX6 (green). Images shown are from 29 somite-stage embryos. Scale bar = 100 μm. **(C′)** Mean expression domain areas of the indicated progenitor markers were measured and normalized to the overall neural tube area. At least four sections per E9.5/25–29 somite stage embryo per genotype were analyzed (gray dots). Pink (OLIG2) and green (PAX6) dots correspond to sections represented in C. **(C″)** Percent OLIG2 or PAX6-positive nuclei were counted by an automated method and normalized to the total DAPI stained nuclei count within neural tubes of each genotype. **(D)** Boxplots of the D/V positions of the indicated progenitor domains relative to neural tube length are shown. The floor plate midline is set to zero. Box tops indicate dorsal boundaries and box bottoms indicate ventral boundaries. Significance was determined by one-way ANOVA. Expression domain analyses were conducted on *n* = 4–5 embryos per genotype and 4–6 sections per embryo. All data were pooled. For all experiments, a P value of <0.05 was considered statistically significant. Significance is denoted as follows: *<0.05, **<0.01, ***<0.001, ****<0.0001, and ns, P > 0.05. Data are represented as mean ± SD.

Having established a cell biological connection between SHH and EP_4_ that stabilizes PC length for optimal downstream signaling, we next sought to determine whether EP_4_ loss altered ciliary length to impact SHH signaling in vivo. Thus, we analyzed developing neural tubes of *Ep4*^*−/−*^ mice to assess PC length and evaluate SHH-regulated ventral neural tube cell fate specification. *Ep4*^*−/−*^ mice are present at normal Mendelian ratios in utero but die at birth due to heart failure resulting from highly penetrant patent ductus arteriosus (Segi et al., 1998). Thus, we collected E9.5/25-29 somite stage wild type, *Ep*_*4*_^*+/−*^ and *Ep*_*4*_^*−/−*^ embryos to perform scanning electron microscopy for visualization of apical PC of cells lining the neural tube lumen (Fig. 6 A). Measurement of apical PC revealed that *Ep4*^*+/+*^ and *Ep4*^*+/−*^ embryos had similar average ciliary lengths, but the average PC length observed in *Ep4*^*−/−*^ embryos was significantly reduced (Fig. 6, A and B). Whereas PC in control neural tubes averaged ∼0.9 µm, cilia in knockout embryos showed an ∼30% length reduction to an average of ∼0.6 µm. Thus, EP_4_ contributes to PC length control in the developing mouse neural tube.

During neural tube development, SHH signals in a graded manner from the notochord and neural tube floor plate to instruct transcriptional programs that establish ventral neural tube cell fates (Ericson et al., 1997; Patten and Placzek, 2000; Ribes and Briscoe, 2009). To test whether EP_4_ loss led to the alteration of SHH-dependent neural tube progenitor domain specification, we examined the expression of the ventral SHH transcriptional target *Olig2*, which is activated by high SHH, and the intermediate fate marker *Pax6* (Fig. 6 C) (Ericson et al., 1997; Ribes and Briscoe, 2009). Quantification of ventral progenitor domain areas in E9.5/25-29 somite stage knockout and littermate control neural tubes using both manual (Fig. 6 C′) and automated (Fig. 6 C″) methods revealed that EP_4_ loss led to a reduction in the *Olig2*-positive motor neuron progenitor cell population (Fig. 6, C–C″). This indicates a reduced SHH signaling response in *Ep4*^*−/−*^ mice. Although the *Pax6*-expressing progenitor domain did not significantly change in size, it shifted ventrally coincident with the reduction of the *Olig2*-positive progenitor population (Fig. 6, C′ and D). Taken together with the above, these results suggest that EP_4_ loss shortens average PC length and compromises SHH-directed cell fate specification in vivo.

## Discussion

PC provide essential contributions to intercellular communication by housing numerous cell surface receptors and their downstream effectors (Hilgendorf et al., 2024; Mukhopadhyay and Rohatgi, 2014). Consistent with this functionality, the SHH pathway signal transducer SMO localizes to the ciliary membrane and communicates with GLI transcriptional effectors as they cycle through the PC (Arensdorf et al., 2016; Kong et al., 2019). We previously reported that cPLA_2_α, which produces the PGE_2_ precursor arachidonic acid, is a direct target of SMO-activated Gβγ. Arachidonic acid drives a feed-forward signal that promotes SMO ciliary enrichment and downstream communication with GLI (Arensdorf et al., 2017). Herein, we reveal that SHH-stimulated production of arachidonic acid also fuels PGE_2_ production and secretion to activate EP_4_ for PC length homeostasis and SHH signaling competency. This activity, along with the documented ability of noncanonical SHH signaling to stimulate primary ciliogenesis (Akhshi and Trimble, 2021), suggests that the SHH pathway has evolved multiple avenues by which it can ensure PC function for a robust signal response.

**Figure 7.**
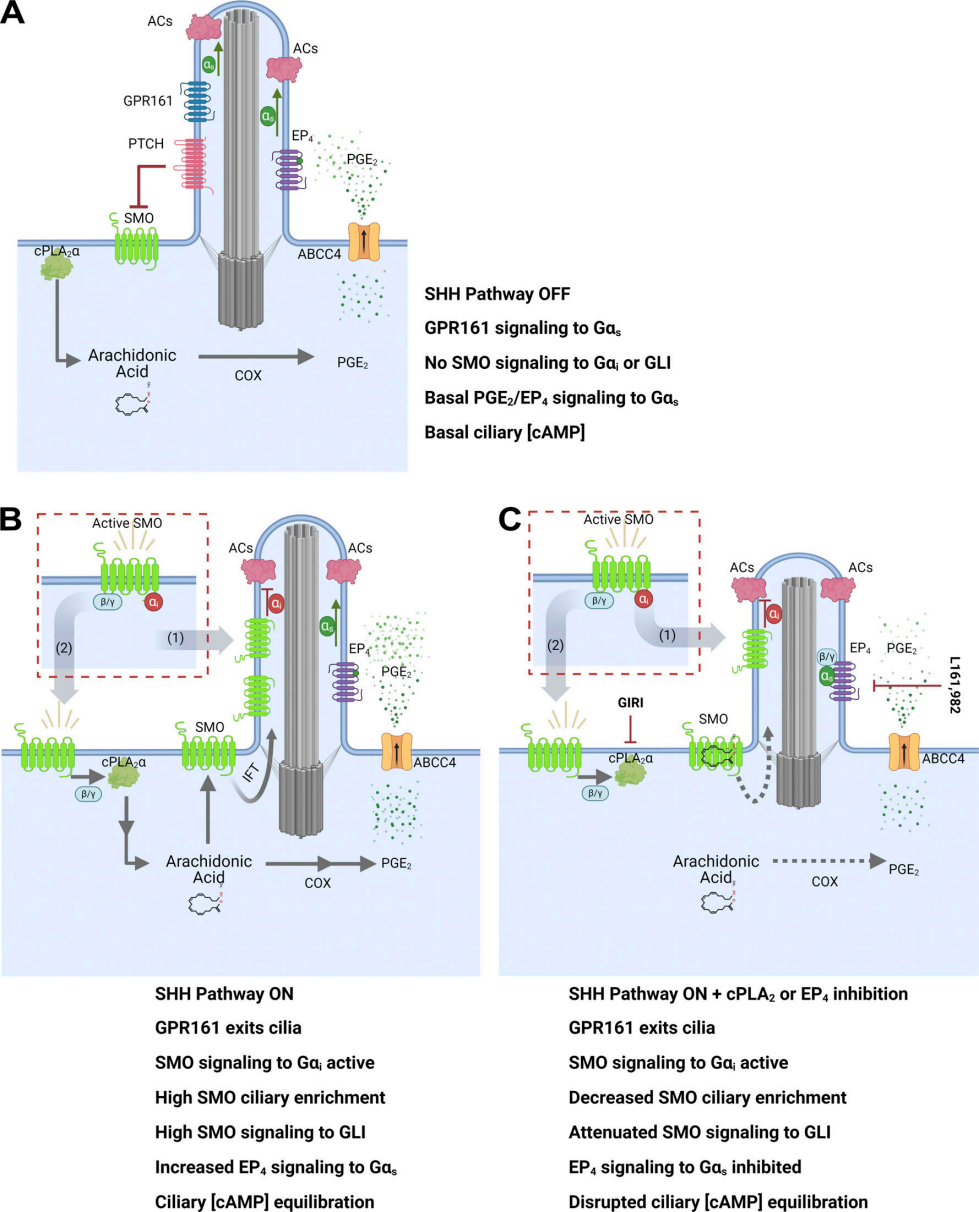
**A model for SHH-to-EP4 signal crosstalk. (A)** In the absence of SHH, the SHH receptor PTCH prevents ciliary accumulation and signaling of the GPCR SMO. Ciliary cAMP is maintained at a level sufficient to ensure anterograde IFT and ciliary length homeostasis through basal PGE_2_ secretion and EP_4_-Gα_s_ activation. Gα_s_-coupled GPR161 is present in the PC. **(B)** SHH binding to PTCH leads to SMO derepression and GPR161 ciliary exit. SMO activates Gα_i_βγ to increase the production of arachidonic acid by cPLA_2_α. Arachidonic acid is metabolized to increase PGE_2_ secretion for enhanced EP_4_-Gα_s_ activation. This ensures ciliary cAMP levels remain sufficiently high in SHH-stimulated cells to maintain anterograde IFT and prevent ciliary shortening. **(C)** Inhibition of cPLA_2_α or EP_4_ reduces SHH-to-EP_4_ signal crosstalk, slows IFT, reduces primary cilium length, and blunts SMO ciliary accumulation and high-level signaling.

A key contribution of PC to the regulation of SHH pathway activity is to facilitate inhibitory GLI transcription factor phosphorylation by PKA in the absence of ligand and allow for a rapid halt of this activity in the presence of SHH (Kong et al., 2019; Umberger and Ogden, 2021). To pause PKA phosphorylation of GLI, SHH must lower ciliary cAMP levels. Accordingly, SHH signaling stimulates ciliary accumulation of Gα_i_-coupled SMO and instructs PC exit of Gα_s_-coupled GPR161 (Fig. 7, A and B). Although favorable for GLI stabilization, these responses risk depleting cilia of cAMP, which can slow IFT and destabilize PC length homeostasis. Consistent with this scenario, we and others have observed that SHH stimulation of cultured murine fibroblasts or IMCD3 cells can trigger modest reductions in ciliary length that occasionally reach statistical significance (this study and Gomez et al. [2022]). However, SHH-stimulated IMCD3 PC shortening does not exceed ∼12% unless SHH-EP_4_ crosstalk is blocked, whereupon length reductions are consistently significant and range from ∼25 to 40%. Pronounced PC shortening following genetic or pharmacological EP_4_ signal disruption correlates with slowing of anterograde IFT and a reduced SHH signal response (Fig. 7 C). We therefore propose that SHH-EP_4_ crosstalk evolved to avoid these complications by equilibrating PC cAMP through EP_4_-Gα_s_ activation of ciliary AC. Notably, SHH simulation leads to phosphorylation of the intracellular carboxyl-terminal tail of SMO by GPCR Kinases (GRK2/3) to expose a PKA inhibitory domain (PKI) that binds PKA to prevent GLI phosphorylation (Arveseth et al., 2021; Happ et al., 2022). We speculate this may be a mechanism by which GLI stabilization is maintained in SHH-stimulated cells following EP_4_ activation and cAMP re-elevation.

Consistent with high PC cAMP and increased PKA activity favoring GLI repression, genetic loss of the ciliary AC chaperone *Ankmy2*, which prevents localized production of ciliary cAMP, triggers SHH-independent GLI accumulation and ectopic target gene induction in vivo (Somatilaka et al., 2020). Curiously, defective ciliogenesis was not observed in *Ankmy2* knockout animals, and length reduction was not noted in NIH-3T3 cell systems used in the published report. We do not know the reason for the discrepancy between our results and theirs but speculate that differences in sample size or ciliary length measurement techniques may be contributing factors. Furthermore, PC shortening following cAMP dysregulation is subtle in NIH-3T3 cells, as demonstrated by the comparatively smaller length reductions we observed in NIH-3T3 versus IMCD3 cells following ANKMY2 loss or ciliary AC reduction. Nevertheless, both studies agree that *Ankmy2* knockout cells are compromised in their ability to induce SHH-stimulated transcriptional responses. This suggests that the ability to precisely tune ciliary cAMP levels up or down is essential for optimal control of downstream SHH pathway activity. Consistent with this notion, we found that knockout of individual ACs reduced ciliary occupancy of other ACs and also blunted SMO accumulation along the length of the cilium. Both situations correlated with reduced target gene activation. Notably, loss of AC5, AC6, or ANKMY2 led to SMO accumulation near the PC base of SHH-stimulated cells in both the absence and presence of PGE_2_, suggesting that depleting cilia of specific ACs stalls anterograde SMO IFT. This may indicate that specific ciliary cAMP “zones” generated by distinct ACs along ciliary membrane work together to promote IFT or regulate SMO signaling to GLI following SHH pathway activation. An alternative explanation is that rapid cAMP elevation that occurs through combined activity of all ciliary ACs may be needed to stimulate IFT following SHH pathway induction. Future studies are needed to evaluate these possibilities and determine the precise molecular mechanism(s) by which cAMP modulates IFT to promote SHH-stimulated SMO ciliary enrichment and downstream signal transduction.

Going forward, it will also be important to evaluate how specific signaling contexts influence the impact of cAMP on anterograde IFT and PC length stability. This is highlighted by a recent study that evaluated the use of optogenetic tools to modulate ciliary cAMP levels. It was reported that stimulation of PC-localized light-activated phosphodiesterase (LAPD), which is predicted to break down ciliary cAMP upon blue light exposure, did not reduce IMCD3 PC length (Hansen et al., 2020). This may indicate that reduced PC cAMP concentration is more detrimental to ciliary length regulation in the context of active signaling than it is when the PC is not engaged in a specific ligand response. Consistent with this hypothesis, we observed that ciliary length reductions triggered by inhibition of PGE_2_/EP_4_ signaling were exacerbated by SHH stimulation. It is also important to note that the LAPD used in the above-referenced study is activated by light stimulation at a wavelength that precludes concurrent use of PC cAMP biosensors (Hansen et al., 2020). Thus, PC cAMP could not be specifically evaluated following LAPD activation or at the time of ciliary length evaluation. We speculate that ciliary length may have shortened at earlier time points following LAPD activation or that compensatory ciliary AC activity was initiated to stabilize PC cAMP and length by the time cilia were measured.

Notably, the capacity of GPCR signaling to affect PC length behavior through cAMP modulation is not limited to SMO or EP_4_. Activation of dopamine-stimulated DRD1-Gα_s_ signaling elongates cilia while activation of DRD2-Gα_i_ or MCHR1-Gα_i_ can shorten PC (Alhassen et al., 2022; Macarelli et al., 2023; Miyoshi et al., 2014; Spasic and Jacobs, 2017). These observations suggest that ciliary length modulation may be a common outcome of PC-localized GPCR activation. Although we do not propose that distinct SHH signaling thresholds are achieved at specific PC lengths, ciliary elongation resulting from genetic or pharmacological manipulation of actin polymerization has been demonstrated to amplify SMO signal output (Drummond et al., 2018). As such, we suggest that diminution of SHH pathway activity that occurs following EP_4_ signal disruption can be attributed primarily to PC length reduction and not to generalized ciliary dysfunction resulting from EP_4_ inactivity. Consistent with this hypothesis, a growing body of evidence demonstrates that distinct cell types have unique PC length ranges that, when altered, can impact signaling capacity (reviewed in Macarelli et al. [2023]). It is not yet clear how PC length affects signal output across cell types, but potential mechanisms include altering the sensitivity of cilia to extracellular cues, shifting receptor density along the ciliary membrane, or changing membrane receptor composition (Besschetnova et al., 2010; Macarelli et al., 2023).

Intriguingly, previously published work suggests that Gα_s_-coupled ciliary GPCRs in addition to EP_4_ may be activated in conjunction with SHH. Gα_s_-coupled D1R and 5-HT_6_ receptors also localize to PC and are sensitized to activation by their cognate ligands following SMO stimulation (Domire et al., 2011; Jiang et al., 2019). Whether these effects result from direct communication between SMO and other GPCR classes, or from ciliary GPCRs tuning their activity to fluctuations in PC cAMP concentration is not yet known. However, work in *Drosophila* suggests that GPCR signal crosstalk to raise cAMP downstream of Hedgehog (Hh) pathway induction may be conserved. In *Drosophila*, Hh-activated Smo stimulates Gα_i_ to lower cAMP and halt PKA-regulated truncation of the GLI ortholog Cubitus interruptus (Ci) (Ogden et al., 2008). However, PKA phosphorylation of *Drosophila* Smo is required for high-level signaling, suggesting that Gα_i_-mediated cAMP lowering could compromise Hh pathway induction (Jia et al., 2004). A potential mechanism by which Smo solves this problem was revealed by the discovery that Smo signaling promotes Gα_s_ activation that enhances pathway responsiveness to low-threshold Hh stimulation (Praktiknjo et al., 2018). It is not yet clear whether Gα_s_ activation downstream of Hh occurs through Smo signal bias or by Smo crosstalk with Gα_s_-coupled GPCR signaling partners. Nevertheless, the observation that Smo can lead to Gα_s_ activation in flies supports an evolutionarily conserved ability of Hedgehog family members to affect exquisite control of cAMP modulators to ensure appropriate downstream signaling activity. The model we propose (Fig. 7) highlights the complex relationship between SHH signaling and cAMP modulation and attempts to reconcile the seemingly incompatible pathway requirements of lowering cAMP to block GLI repressor formation while maintaining sufficient ciliary cAMP for functional ciliary trafficking, length homeostasis, and downstream SMO signal transduction.

## Materials and methods

### Reagents

All reagents, kits, primers, plasmids, mice, cell lines, antibodies, and software used in the study are detailed in Table 1, along with their source identification numbers.

**Table 1.** Reagents and resources

Reagent or Resource	Source	Identifier
Antibodies		
Smoothened	Santa Cruz Biotechnology	sc-166685, RRID:AB2239686
Acetylated α-Tubulin	Cell Signaling	5335S, RRID:AB_0544694
ARL13B	Antibodies Incorporated	75-287, RRID:AB_2341543
ARL13B	BiCell Scientific	ARL2L1
EP_4_	Santa Cruz Biotechnology	sc-55596, RRID:AB_2174775
AC3 (IF)	Proteintech	19492-1-AP RRID:AB_10638445
AC3 (WB)	Abcam	352098
AC5 (WB)	Life Technologies	BS-3922R RRID:AB_10857032
AC5/6 (IF)	FabGennix	PAC-501AP
AC6 (WB)	Proteintech	14616-1-AP-20UL RRID:AB_2242343
ANKMY2 (WB)	Sigma-Aldrich	HPA067100 RRID:AB_2685777
PAX6	Developmental Studies Hybridoma Bank	PAX6-s RRID:AB_528427
OLIG2	Millipore	AB9610, RRID:AB_570666
GPR161	Saikat Mukhopadhyay	Pal et al. (2016)
ABCC4	John Schuetz	Wijaya et al. (2020)
Kinesin	Abcam	ab167429 RRID:AB_2715530
AlexaFluor 488	Life Technologies	A11029 (mouse), RRID:AB_2534088; A11034 (rabbit), RRID:AB_2576217
AlexaFluor 555	Life Technologies	A21424 (mouse), RRID:AB_141780; A21429 (rabbit), RRID:AB_2535850
AlexaFluor 647	Life Technologies	A21236 (mouse), RRID:AB_2535805; A21245 (rabbit), RRID:AB_2535813
Chemicals, peptides, and recombinant proteins		
Arachidonic acid	Cayman Chemical Company	90010-50
Giripladib	WuXi Custom Synthesis	Duvernay et al. (2015)
L161,982	Tocris Chemicals	2,514
Dibutyryl-cAMP (dBcAMP)	MedChem Express	HY-B0764
Sonidegib (LDE225)	MedChem Express	HY-16582
Prostaglandin E_2_ (PGE_2_)	Sigma-Aldrich Chemicals	P5640
Celecoxib (Coxib)	Cayman Chemical Company	10008672
Forskolin (FSK)	Sigma-Aldrich Chemicals	F6886-10MG
SAG	Selleck Chemical Company	S7779-2MG
Lipofectamine 3000	Thermo Fisher Scientific	L3000008
Lipofectamine RNAiMAX	Thermo Fisher Scientific	13778150
IBMX	Sigma-Aldrich	I5879-1G
DMSO	Sigma-Aldrich	D2650
Hygromycin	Life Technologies	10687010
Commercial assay kits		
Prostaglandin E_2_ parameter assay kit	R&D Systems	KGE004B
BCA protein assay kit	Thermo Fisher Scientific Pierce	23227
Flp-In complete system	Thermo Fisher Scientific	K601001
RNeasy plus mini kit	Qiagen	74136
High-capacity cDNA reverse transcription kit	Applied Biosystems	4368813
Direct cAMP ELISA kit	Enzo Life Sciences	ADI900066
Ratio-metric cilia-targeted cADDis cAMP	Montana Molecular	D0211G
PowerUp SYBR green master mix	Applied Biosystems	A25742
PowerPlex fusion system	Promega	DC2402
MycoAlert plus	Lonza	LT07-710
Oligonucleotides		
*Ptch1* Forward: 5′-TCA​CCT​CCA​TCA​GCA​ATG​TC-3′	Thermo Fisher Scientific	N/A
*Ptch1* Reverse: 5′-GAA​TAC​CAC​CAC​CAC​AGC​AG-3′	Thermo Fisher Scientific	N/A
*Gli1* Forward: 5′-CCA​AGC​CAA​CTT​TAT​GTC​AGG​G-3′	Thermo Fisher Scientific	N/A
*Gli1* Reverse: 5′-AGC​CCG​CTT​CTT​TGT​TAA​TTT​GA-3′	Thermo Fisher Scientific	N/A
*Adcy3* Forward: 5′-GCT​TTC​TTT​GTC​TTC​TCC​TTC-3′	Thermo Fisher Scientific	N/A
*Adcy3* Reverse: 5′-ATG​ATA​GCA​CAC​AGG​TAG​AG-3′	Thermo Fisher Scientific	N/A
*Adcy5* Forward: 5′-GTC​TCA​GAA​AGT​GGC​AAT​TC-3′	Thermo Fisher Scientific	N/A
*Adcy5* Reverse: 5′-CAT​TCA​GGT​AGT​TGA​GTG​TG-3′	Thermo Fisher Scientific	N/A
*Anmky2* Forward: 5′-GCT​GCC​AAA​ACA​TAA​GAG​ACA​G-3′	Thermo Fisher Scientific	N/A
*Anmky2* Reverse: 5′-CTT​CTT​TCT​CCC​TTC​CAC​AG-3′	Thermo Fisher Scientific	N/A
*Ppia* Forward: 5′-AGC​ACT​GGA​GAG​AAA​GGA​TT-3′	Thermo Fisher Scientific	N/A
*Ppia* Reverse: 5′-ATT​ATG​GCG​TGT​AAA​GTC​ACC​A-3′	Thermo Fisher Scientific	N/A
*Btf3* Forward: 5′-GAA​CAA​CAT​CTC​TGG​TAT​TGA​AGA-3′	Thermo Fisher Scientific	N/A
*Btf3* Reverse: 5′-AAT​GGT​GAA​GGT​GTT​TGC​TG-3′	Thermo Fisher Scientific	N/A
NGS primer Adcy3 forward: 5′-CTA​CAC​GAC​GCT​CTT​CCG​ATC​TGG​TGT​ACT​GGG​CTC​CTC​AGG​GCT​AG-3′	Integrated DNA Technologies	N/A
NGS primer Adcy3 reverse: 5′-CAG​ACG​TGT​GCT​CTT​CCG​ATC​TAC​ACA​CCA​TCG​CTC​CTG​AGT​CTC-3′	Integrated DNA Technologies	N/A
Adcy5 primer forward: 5′-CTA​CAC​GAC​GCT​CTT​CCG​ATC​TTC​TGG​CAG​GCA​GCT​TAC​TCC​AAG​GA-3′	Integrated DNA Technologies	N/A
NGS Adcy5 primer reverse: 5′-CAG​ACG​TGT​GCT​CTT​CCG​ATC​TGA​AAG​CCA​GGA​GCC​CCG​GAG​CAG​AG-3′	Integrated DNA Technologies	N/A
NGS primer Adcy6 forward: 5′-CTA​CAC​GAC​GCT​CTT​CCG​ATC​TTG​ATC​TAG​GCA​GGC​TGG​GAT​CTG​AA-3′	Integrated DNA Technologies	N/A
NGS primer Adcy6 reverse: 5′-CAG​ACG​TGT​GCT​CTT​CCG​ATC​TAC​CTA​CAA​GGC​ACT​GGG​GGT​GAA​GGC-3′	Integrated DNA Technologies	N/A
NGS primer Ankmy2 forward: 5′-CTA​CAC​GAC​GCT​CTT​CCG​ATC​TTG​TGT​GTA​TGT​GTC​ATG​AAG​ATG​G-3′	Integrated DNA Technologies	N/A
NGS primer Ankmy2 reverse: 5′-CAG​ACG​TGT​GCT​CTT​CCG​ATC​TCT​GCC​CGG​CTG​TAA​TAG​CTT-3′	Integrated DNA Technologies	N/A
sgRNA spacer Adcy3: 5′-CUA​CCU​GUG​UGC​UAU​CAU​CG-3′	Integrated DNA Technologies	N/A
sgRNA spacer Adcy5: 5′-AUU​GUU​ACU​ACU​GCG​UCU​CG-3′	Integrated DNA Technologies	N/A
sgRNA spacer Adcy6: 5′-CCA​ACA​GCU​CGG​UGC​UAA​CG-3′	Integrated DNA Technologies	N/A
sgRNA spacer Ankmy2: 5′-CUC​AUG​UUU​GCU​GCG​CUU​UC-3′	Integrated DNA Technologies	N/A
*Adcy3* siRNA	Thermo Fisher Scientific	Assay ID: S98223
*Adcy5* siRNA	Thermo Fisher Scientific	Assay ID: S104765
*Anmky2* siRNA	Thermo Fisher Scientific	Assay ID: MSS235827
Experimental models: Cell lines		
IMCD3	ATCC	CRL-2123
IMCD3 Flp-In cells	Maxence Nachury Lab	Ye et al. (2013)
IMCD3 *Adcy3*^*−/−*^ cells	This paper	N/A
IMCD3 *Adcy5*^*−/−*^ cells	This paper	N/A
IMCD3 *Adcy6*^*−/−*^ cells	This paper	N/A
IMCD3 *Ankmy2*^*−/−*^ cells	This paper	N/A
IMCD3 EF1α-cyto-bPAC-GFP	This paper	N/A
IMCD3 EF1α-Arl13b-Cilia-bPAC-GFP	This paper	N/A
NIH-3T3	ATCC	CRL-1658
*Abcc4*^*−/−*^ NIH-3T3	John Schuetz lab	Wijaya et al. (2020)
*Ep4*^*−/−*^ mouse embryonic fibroblast	This paper	N/A
Wild type mouse embryonic fibroblast	This paper	N/A
Experimental models: Mouse model		
*Ep4*^*−/−*^ mice	Elaine Shelton	Segi et al. (1998)
Recombinant DNA		
pgLAP5-EF1α dTATA-bPAC-GFP	Gift from Reiter lab	Truong et al. (2021)
pgLAP5-EF1α dTATA-Arl13b-bPAC-GFP	Gift from Reiter lab	Truong et al. (2021)
pEF1α-mIFT88-EGFP	Addgene	149697
pCDNA-DEST47-Arl13b-GFP	Addgene	40872
pCDNA3-EGFP	Addgene	13031
PTGER4-Tango	Addgene	66486
5HT6-mCherry-G-GECO1.0	Addgene	47500
pKK44-ABCC4	Gift from Schuetz lab	Wijaya et al. (2020)
pCDNA-h-mmPtch1-B-HA-GFP	Addgene	120903
Software and algorithms		
GraphPad prism v. 10	GraphPad Software	http://www.graphpad.com/
ImageJ/Fiji	Schindelin et al. (2012)	https://imagej.net/software/fiji/
LAS X	Leica	https://www.leica-microsystems.com/products/microscope-software/details/product/leica-las-x-ls/
JACoP	Open Source	https://imagej.nih.gov/ij/plugins/track/jacop2.html
KymographDirect v. 2.0	Open Source	https://sites.google.com/site/kymographanalysis/
NIS-elements viewer v. 4.50.00	Nikon	https://www.microscope.healthcare.nikon.com/products/software/nis-elements/viewer
Inkscape	Open Source	https://inkscape.org/
StarDist	Stevens et al. (2022)	https://github.com/stardist/stardist
CRIS.py (v2.0)	Connelly and Pruett-Miller (2019)	https://github.com/patrickc01/CRIS.py

### Chemicals

L161,982, di-butyryl cAMP (dBcAMP), Sonidegib (LDE-225), and Celecoxib (Coxib) were reconstituted in DMSO and stored at −20°C. Arachidonic acid was reconstituted and stored in ethanol at −20°C. Prostaglandin E_2_ (PGE_2_) and Forskolin (FSK) were reconstituted in ethanol and stored at 4°C. GIRI was synthesized by WuXi and stored according to published procedures (Duvernay et al., 2015; Thotala et al., 2013).

### Mammalian cell culture

Wild-type and Flp-In IMCD3, *Adcy3*^*−/−*^, *Adcy5*^*−/−*^ *Adcy6*^*−/−*^, and *Ankmy2*^*−/−*^ cells were cultured in Dulbecco’s Modified Eagle Medium (DMEM): F12 supplemented with 10% fetal bovine serum (FBS) and 1% penicillin/streptomycin solution (pen/strep, Gibco). Wild-type MEFs, NIH-3T3, *Ep4*^−/−^ MEF, and *Abcc4*^*−/−*^-NIH-3T3 cells were cultured in DMEM supplemented with 10% fetal calf serum, 2 mM *L*-glutamine, 1 mM sodium pyruvate, 1× non-essential amino acids, and 1% pen/strep. Cells were passaged routinely with 0.25% Trypsin/EDTA solution and maintained under standard incubation conditions (5% CO_2_, 37°C) in a humidified incubator. All cell lines were routinely tested for mycoplasma contamination. To promote ciliogenesis, cell lines were serum starved for 2 h in serum-free media (DMEM or DMEM: F12, supplemented with 0.1 mM nonessential amino acids, 2 mM *L*-Glutamine, 1 mM sodium pyruvate, and 1% pen/strep). After starvation, media was replaced with low serum (0.5%) media and cells were incubated for 18 or 36 h.

### Cell line generation

IMCD3 gene knockout pools were generated using CRISPR-Cas9 technology in the Center for Advanced Genome Engineering at St. Jude Children’s Research Hospital. Briefly, 1 million IMCD3 cells (ATCC) were transiently transfected with precomplexed ribonuclear protein (RNPs) consisting of 300 pmol of each Alt-R sgRNA (IDT) and 100 pmol of 3X NLS *Sp*Cas9 protein (St. Jude Protein Production Core) via nucleofection (Lonza, 4D-Nucleofector X-unit) using solution P3 and program DS-137 in a large (100 μl) cuvette according to the manufacturer’s recommended protocol. 3 days post nucleofection, cell pellets of ∼100,000 cells were lysed and used to generate gene specific amplicons that were sequenced via targeted next generation sequencing (NGS) as previously described (Narina et al., 2023). NGS data was processed using CRIS.py to report total editing for each pool (Connelly and Pruett-Miller, 2019). After expansion, the final pools were authenticated using the PowerPlex Fusion System (Promega) performed at the SJCRH Hartwell Center and tested negative for mycoplasma by the MycoAlertPlus Mycoplasma Detection Kit (Lonza). Knockout pools were further verified by Western blot. sgRNA spacer sequences and relevant primers are listed in Table 1.

EP_4_ knockout cells were generated from *Ep4*^*−/−*^ embryos (Segi et al., 1998). Embryos (13.5 dpc) were freshly isolated and placed in individual 10-cm culture dishes where they were eviscerated. Heads were removed and used for genotyping to identify homozygous null embryos and wild-type littermates using conventional PCR. Embryo bodies were transferred to 15-ml conical tubes containing 5 ml fresh PBS and dissociated by aspirating through a 16-gauge needle into a 10 ml syringe and expelling the contents. Warm media (DMEM + 10% FBS + 1% pen/strep) was then added to each conical tube to bring the total volume to 10 ml. This solution was transferred to a 15 cm culture dish containing 25 ml additional media and placed in a 37°C incubator. After 24 h, media and non-adherent tissue pieces were removed and fresh media was added. Cells were grown to 80–90% confluence, after which they were passaged and frozen down after passage number two. Generation of *Abcc4*^*−/−*^ NIH-3T3 cells has been described previously (Wijaya et al., 2020).

### Generation of optogenetic bPAC cells

IMCD3 cell lines stably expressing Cyto-bPAC-GFP and Cilia-bPAC-GFP were generated using the Flp-In system following manufacturer’s instructions (Thermo Fisher Scientific). IMCD3 Flp-In cells (Ye et al., 2013) were transfected using Lipofectamine 3000 with the appropriate plasmids and selected with 40 mg/ml hygromycin (Gibco). Single colonies were expanded, and protein expression was confirmed by fluorescence microscopy for GFP.

### Immunofluorescence and ciliary length measurement

Cells were plated onto coverslips (Corning) and pretreated with either vehicle or drug in serum-free media. After 2 h, media was changed to low serum media containing vehicle or drug and SHH or control conditioned media (100 µl/ml) and then incubated for ∼18 h. After incubation, cells were washed with PBS and then fixed in 4% paraformaldehyde for 12 min. Cells were then washed with wash buffer (PBS with 0.1% Triton X-100) three times for 5 min and incubated in blocking buffer for 60 min at room temperature (PBS with 2% BSA, 0.1% Triton X-100, 1% goat serum). Primary antibody incubations were performed overnight at 4°C. The following antibodies and dilutions were used: anti-SMO (1:500; Santa Cruz), anti-acetylated α-tubulin (1:1,000; Cell Signaling), anti-ARL13B (1:500; Antibodies Incorporated or 1:1,000; BiCell Scientific), anti-EP_4_ (1:100; Santa Cruz), anti-GPR161 (1:500), anti-AC5/6 (1:75; FabGennix), and anti-AC3 (1:200; Proteintech) diluted in blocking buffer. Secondary antibody incubations were performed with AlexaFluor 488, 555, or 647 conjugated secondary antibodies (1:1,000; Life Technologies) and DAPI for 60 min at room temperature. Following antibody incubation, samples were washed three times with the wash buffer followed by a PBS rinse prior to mounting with ProLong Diamond (Life Technologies). Images were collected using Leica TCS SP8 STED 3X confocal microscope with a 63× oil-immersion objective and processed using LAS X (Leica). For all immunofluorescence experiments, multiple cells (≥100) were examined over at least two independent experiments. Representative images are shown.

For ciliary length measurement, a maximum projection of the acquired Z stack images was created. Each cilium was manually traced from the base to the tip using the line profile tool in the LAS X software. To ensure an unbiased approach, subsets of PC length measurements were evaluated by a lab member who was blinded to experimental condition. For quantification graphs, all data are included. For microscopy images, representative cilia are shown.

### Quantification of ciliary length and fluorescence intensities

Quantification of ciliary lengths and SMO, PTCH1, ARL13B, and GPR161 ciliary accumulation values were calculated from at least three independent experiments with at least 100 cilia per condition per experiment. A maximum fluorescence intensity projection from a sum of at least 10 slices was generated before length and ciliary intensity quantifications. SMO, PTCH1, ARL13B, and GPR161 ciliary signal intensities were normalized to acetylated α-tubulin or ARL13B signal. At least three randomly chosen fields of view were selected per condition per experiment, and all cilia within the field were quantified using LAS X. For ciliary SMO/PTCH1/GPR161/ARL13B signal intensity determination, each cilium surface area was traced from the base to the tip using spline profile function. The average fluorescence measurement adjacent to the cilium was subtracted from ciliary fluorescence as background. The intensity values along the cilium were exported and analyzed via GraphPad Prism. Colocalization of AC3 and AC6 with the PC marker ARL13B was carried out using the ImageJ plugin JACoP. Manders’ coefficients estimate the co-occurrence fraction of a fluorescent signal on one channel (ARL13B) with a fluorescent signal of another channel (AC3/AC5/6). Manders’ coefficients range from 0 to 1.

Percent EP_4_ positive cilia were calculated by manually scoring EP_4_ signal overlapping with the acetylated α-tubulin ciliary signal. Approximately, 75 cilia were scored over two independent experiments and all data were pooled.

Statistical analyses were performed using Prism 10 (GraphPad Software). Results are presented as mean ± standard deviation. For comparisons between two groups, a two-tailed unpaired *t* test was used unless otherwise specified. For multiple group comparisons, either one-way or two-way (depending on the number of variables) ANOVA followed by multiple comparison post-hoc testing was performed as indicated using Prism 10. Significance is depicted as *P < 0.05, **P < 0.01, ***P < 0.001, ****P < 0.0001, ns = not significant.

### Immunoblotting

For Western blotting, SDS-PAGE samples were run on 4–15% Tris-glycine SDS-PAGE gels (Bio-Rad) and transferred onto Immobilon-P PVDF (Millipore) using Tris/Glycine/SDS Buffer (Bio-Rad) at 100 V for 1 h. Membranes were blocked with 5% milk in Tris-buffered saline with 0.1% Tween-20 (TBST) for an hour at room temperature. Antibody dilutions used were as follows anti-EP_4_ (1:500; Santa Cruz), anti-ABCC4 (1:500), anti-AC6 (1:500; Proteintech), anti-AC3 (1:500; Abcam), anti-AC5 (1:500; Life Technologies), anti-ANKMY2 (1:1,000; Sigma-Aldrich), and anti-Kinesin (1:5,000; Abcam). Appropriate HRP-conjugated secondary antibodies (Jackson Immuno) were incubated for 1 h at RT at a 1:5,000 concentration. Blots were developed using an Odyssey Fc (Li-Cor) with ECL Prime (GE).

### DNA transfection

Cells including IMCD3 WT, *Adcy3*^*−/−*^, *Adcy5*^*−/−*^, *Adcy6*^*−/−*^, *Ankmy2*^*−/−*^, WT MEF, *Ep4*^*−/−*^, and *Abcc4*^*−/−*^ were seeded onto coverslips in six-well plates and transfected with *pCDNA3-eGFP*, *pCDNA3-Ptch1-GFP*, *pCDNA3-Arl13B-GFP*, *Tango-Ep4*, or *pKK44-Abcc4* (2 μg each) using Lipofectamine 3000 as per the manufacturer’s protocol. The next day, cells were serum-starved in serum-free media with or without drug treatment for 2 h. Media was replaced with low-serum media containing the indicated drug or vehicle control and SHH or control conditioned media (100 µl/ml) and then cells were incubated for ∼18 h. After incubation, coverslips were processed for the immunostaining protocol as described above.

### siRNA transfection

Transfections using Lipofectamine RNAiMAX (Thermo Fisher Scientific) were performed following the manufacturer’s protocol. Briefly, NIH-3T3 cells were plated in six-well dishes at a density of 100,000 cells per well. The following day, cells were treated with 25 pmol of either scrambled or *Adcy3*, *Adcy5*, or *Ankmy2* siRNA. Cells were analyzed 48 h after transfection.

### Optogenetic stimulation

bPAC-expressing IMCD3 Flp-In cells were plated onto coverslips, grown to confluency, and pretreated with either vehicle or L161,982 (10 µM) in serum-free media. After 2 h, the media was changed to low serum media containing vehicle or drug and SHH or control conditioned media (100 µl/ml) and then incubated for ∼18 h. Cells were transferred to a custom-made LED humidified incubator with 5% CO_2_ for continuous blue light 450 nm stimulation at ∼0.4 mW/cm^2^ for 3 h (Zhang et al., 2019). Following this, immunofluorescence staining was performed as described above.

### PGE_2_ ELISA

Cells were cultured in 12-well dishes and pretreated for 2 h with either LDE225 (10 nM) or GIRI (2–4 µM) in serum-free media. Following pretreatment, media was changed to low-serum media containing either vehicle, LDE225, or GIRI plus control or SHH conditioned media, and then incubated for ∼36 h. PGE_2_ levels were determined by ELISA (R&D Systems). The absorbance of each well was measured at 450 nm with correction at 540 nm.

For rescue experiments in *Abcc4*^*−/−*^ cells, cells were seeded in a 12-well culture plate and the following day they were transfected with either *pcDNA3-eGFP* or *pKK44-Abcc4* (1 µg/well) using Lipofectamine 3000. The next day, cells were pretreated for 2 h in serum-free media. Following pretreatment, media was changed to low serum media containing SHH or control conditioned media, and cells were incubated for 36 h and PGE_2_ levels were determined as described above.

### Cellular cAMP measurement

cAMP in cell lysates was measured using the Direct cAMP ELISA kit (Enzo). Cells were seeded in six-well plates at a density of 3.5 × 10^5^ cells per well. The next day, cells were starved for 2 h in serum-free media followed by overnight incubation in low-serum media to promote ciliation. On the day of treatment, cells were treated with 100 μM IBMX diluted in low serum media and scraped into lysis buffer (0.1 M HCl; Enzo). Samples were analyzed per the manufacturer’s protocol. cAMP concentrations were calculated using a four-parameter logistic (4Pl) and normalized to the total protein concentrations, determined by a BCA protein assay (Thermo Fisher Scientific).

### Ciliary cAMP assay

Cells were seeded at 18,000 cells/well in an eight-well chamber slide (Nunc Lab-Tek II Chamber Slide System) and transduced the next day with the ratiometric cilia-targeted cADDis BacMam (Montana Molecular) as per the manufacturer’s recommendation. Cells were infected with 20 µl of BacMam sensor stock in a total of 250 µl of media containing 2 mM sodium butyrate (Montana Molecular) for 30 min at room temperature in the dark followed by 5 h at 37°C. BacMam was removed and replaced with low-serum media containing 1 mM sodium butyrate for 16–24 h with or without vehicle or L-161,982 (10 μM). Prior to imaging, cells were incubated in PBS for 20 min at room temperature. Positive agonist FSK (100 µM) was added 30 s after the recording was initiated, and images were acquired on a Nikon A1R confocal on live cell imaging mode (60×, epifluorescence) every 10 s for 8 min while maintaining the cells in 5% CO_2_ at 37°C on a heated stage (OKOLAB). For experiments with SAG, the signal was recorded for basal conditions followed by incubation with forskolin for 1.5 min and then treated with either SAG (1 μM) or vehicle and recorded every 10 s for 8 min.

For quantification of cAMP, the red fluorescence (ciliary reference marker) and green fluorescence (cAMP sensor) intensities over time were processed on the Nikon NIS-Elements imaging software by defining a mask for each cilium and determining the ratio of green fluorescence to red fluorescence. The data were normalized to the initial time point for each experiment (set to 100%), and the inverse of each value was plotted using GraphPad Prism.

### Quantitative reverse transcriptase polymerase chain reaction (qRT-PCR)

Total RNA was extracted from cells using the RNeasy Mini Kit (Qiagen) according to the manufacturer’s protocol. 1000 ng of RNA was used to synthesize complementary DNA (cDNA) using High-Capacity cDNA Reverse Transcription Kit (Applied Biosystems). qRT-PCR reactions were performed on a QuantStudio 7 Flex PCR machine using PowerUp Sybr Green Master Mix (Applied Biosystems). Corresponding changes in the expression levels of selected genes were calculated using the ΔΔCt method relative to housekeeping genes, *Ppia* and *Btf3* (Arensdorf et al., 2017).

For qRT-PCR analysis, IMCD3 WT, Cyto-bPAC, and Cilia-bPAC cells were seeded in a 12-well plate. The next day, cells were starved in serum-free media for 2 h, subsequently, the media was changed to low-serum media containing SHH-conditioned or control media or SAG (100 nM). After 18 h of incubation, cells were either treated with no light or blue light for an additional 3 h. Following this, RNA isolation and qRT-PCR were carried out as described above.

### IFT88 kymographs

Kymographs for anterograde IFT88 velocities were generated in IMCD3 cells as previously described (Besschetnova et al., 2009). Briefly, 120,000 cells were grown on a coverslip in a 24-well plate. Cells were transfected with IFT88-GFP and 5HT6-mCherry plasmids the following day. Cells were pretreated with vehicle or the EP4 inhibitor L161,982 (10 μM) for 2 h and then switched to low serum media containing L161,982 either in the presence or absence of SHH or control conditioned media and incubated overnight. On the day of the experiment, the coverslips were flipped onto a two-well chamber slide (Nunc Lab-Tek II Chamber Slide System) and the cells were imaged every 200 ms for 30 s on a Nikon A1R confocal microscope with a 60× oil objective and 37°C, 5% CO_2_ heat stage (OKOLAB). The resulting videos were analyzed by ImageJ macros KymographClear and KymographDirect to generate violin plots (Mangeol et al., 2016).

### Mouse models

Data and materials generated from animals were obtained in accordance with SJCRH/IACUC approved protocol 608-100616-10/19 and Vanderbilt protocol M1600016-01. All animal husbandry and procedures were performed in accordance with protocols approved by St. Jude Children’s Research Hospital and Vanderbilt University Institutional Animal Care and Use Committees.

### Mouse immunohistochemistry

*Ep4*^*+/+*^*, Ep4*^*+/−*^, and *Ep4*^*−/−*^ embryos in the C57BL/6 background were harvested and processed for immunohistochemistry at E9.5/25-29 somites. Pregnant dams were harvested, uterine horns removed, and embryos dissected in 1X PBS, then rinsed three times in 1X PBS. Embryos were fixed overnight at 4°C in 4% paraformaldehyde (PFA). The following day, embryos were rinsed three times in 1X PBS and transferred to 30% sucrose to cryoprotect. The following day, embryos were frozen in O.C.T. Compound (Tissue-Tek) on dry ice. Embryos were sectioned transverse at 10 or 15 µm thickness on a Leica Microm CM1950 cryo-stat. Sections were briefly dried, then washed in 1X TBST, then blocked with 2% BSA, 1% goat serum, and 0.1% Triton-X-100 in 1X PBS. Antibodies were diluted in a blocking buffer and incubated overnight in sections at room temperature. The following antibodies and dilutions were used: mouse anti-PAX6 (1:10; DSHB) and rabbit anti-OLIG2 (1:300; Millipore). The primary antibody was removed, sections were washed with 1X TBST three times, and then incubated for 3 h in secondary antibodies (Invitrogen) at a 1:500 dilution. Sections were washed three times in 1X TBST, then rinsed with water, dried, and coverslips were applied with ProLong Gold mounting media with DAPI. Sections were imaged using an HC PL APO 20× NA 0.80 air objective on a Leica DMi8 widefield microscope equipped with a LED epi fluorescence illumination system, channel-specific filters for DAPI, 488, 561, and 647 compatible dyes, and a Hamamatsu Flash4.0 camera. A minimum of four embryos per genotype were analyzed.

All images analyzed are from the cardiac region adjacent to somites 2 and 3. The landmarks used to identify and align cardiac level transverse sections were the branchial pouches, aortic sac, and size/morphology of atria/ventricles. The Kaufman Atlas of Mouse Development Plate 19b images g and h show the region of the cardiac level images that were analyzed for expression domain quantifications (Graham et al., 2015).

### Neural tube progenitor domain quantification

To measure the total areas of OLIG2 and PAX6-positive neuronal progenitor domains, transverse cardiac level sections adjacent to somites 2 and 3 were analyzed by measuring the total progenitor marker expression domain area relative to the entire neural tube area. The area of the neural tube and the area of the progenitor domain were measured using the Segmented Line Tool in ImageJ. Expression domain areas were calculated by measuring the area of the indicated expression domain and normalizing it to the total area of the neural tube in each section analyzed (Hall et al., 2024). Three to five embryos were analyzed per genotype with five to eight sections analyzed per embryo.

To calculate the number of OLIG2 and PAX6 positive nuclei in the neural tube, raw fluorescent images were processed to identify the total number of cells (using DAPI nuclear stain) and the proportion of these nuclei co-occurring with OLIG2 (magenta) or PAX6 (green) signal in each tissue section after manual segmentation of the neural tube. Following upscaling of the raw data by a factor of 2× using Fiji, individual nuclear outlines were detected using StarDist on the fluorescent signal from the DAPI staining (Schindelin et al., 2012; Stevens et al., 2022). The resulting cell masks were used to measure the sum of fluorescent signals in each one of the two other channels (magenta and green). Results from the entire dataset (all conditions combined) were assembled and analyzed as a batch. Briefly, the distribution of the intensity of the fluorescence staining was plotted to evaluate the threshold values between negative and positive cell populations for each staining. These intensity thresholds were then applied across the entire dataset, and the results were formatted into a table reporting the total number of cells, the number of cells for OLIG2 (magenta), and the number of positive cells for PAX6 (magenta) for each neural tube analyzed. The graph is represented as a percentage of the positive nuclei for each domain normalized to the total DAPI positive nuclei in the neural tube of each section analyzed.

To quantify the relative position of OLIG2 and PAX6 expressing domains along the dorsal ventral (D/V) axis, at least three sections per embryo from the cardiac region adjacent to somites 2 and 3 were used. The total neural tube length from the floor plate to the roof plate was measured using the Segmented Line Tool in ImageJ. The length of the individual progenitor domain along the D/V axis was drawn using the Segmented Line Tool in ImageJ noting the distance of the boundary of the domain from the roof plate and from the floor plate to give domain length and position along the D/V axis (Hall et al., 2024). The graph demonstrates the relative position of each domain within the neural tube. The data are presented as mean ± SD, and statistical comparisons were made using one-way ANOVA. GraphPad software was used for statistical analysis. P values <0.05 were considered statistically significant.

### Electron microscopy

For electron microscopy of neural tube PC, samples were fixed in 2.5% glutaraldehyde, 2% paraformaldehyde in 0.1 M cacodylate buffer containing 2 mM MgCl_2_. Following fixation, samples were dissected to expose the interior of the neural tube lumen. Dissected samples were buffer washed, post-fixed in 0.1% aqueous osmium tetroxide for 1 h, washed with ddH_2_O, dehydrated with an ascending ethanol series, and critical point dried in liquid CO_2_ using an Autosamdri 931 (Tousimis). Dried samples were sputter-coated under planetary rotation with 15 nm iridium and imaged in a Thermo Fisher Scientific Teneo scanning electron microscope at 2 kV using the Everhart-Thornley and T1 detectors.

### Statistical analyses

All statistical analyses were performed using Prism 10 (GraphPad Software). Results are presented as mean ± standard deviation. For comparisons between two groups, Student’s *t* test was used. For multiple group comparisons, one-way ANOVA followed by multiple comparison post-hoc testing was performed as indicated using Prism 10. Significance is depicted as *P < 0.05, **P < 0.01, ***P < 0.001, ****P < 0.0001, ns = not significant.

### Online supplemental material

Fig. S1 provides further validation of the effects of ABCC4 knockout on PGE_2_ secretion and primary cilium length and demonstrates that exogenous arachidonic acid (AA) can partially rescue ciliary length reduction following cPLA_2_ inhibition with GIRI. Fig. S2 shows the effect of EP_4_ knockout on PTCH1 and GPR161 ciliary localization. Fig. S3 shows AC protein reduction in knockout cell pools and provides validation for the fluorescent ciliary cAMP sensor used in the study. Fig. S4 shows the effect of AC knockout on PTCH1 and GPR161 ciliary localization. Fig. S5 provides validation of *Adcy* or *Ankmy2 *knockdown in NIH3T3 cells and shows the effect of knockdown on primary cilium length and* Gli1 *transcriptional activation. The effect of cytoplasmic and ciliary cAMP modulation by photoactivatable AC on ciliary length and SHH target gene activation is also provided.

## Supplementary Material

SourceData FS1 is the source file for Fig. S1.

SourceData FS2 is the source file for Fig. S2.

SourceData FS3 is the source file for Fig. S3.

## Data Availability

All data are available from the corresponding author upon request.
