# Supplementary figures and images for: Sonic Hedgehog activates prostaglandin signaling to stabilize primary cilium length
Source: J Cell Biol. 2024 Jun 10;223(9):e202306002. doi: 10.1083/jcb.202306002 (PMC11166601; doi:10.1083/jcb.202306002)

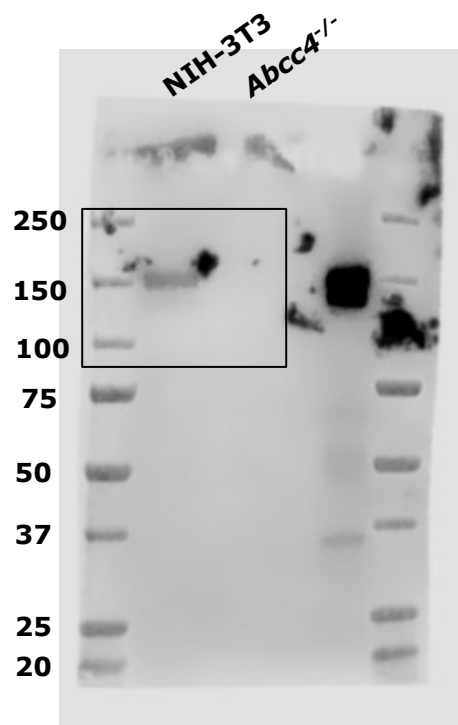

**ABCC<sub>4</sub>**

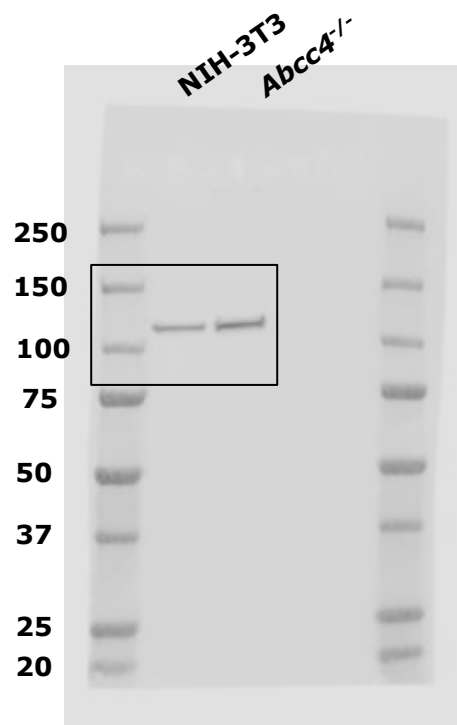

**KINESIN**

Supplement: SourceData FS1 — is the source file for Fig. S1. [file JCB_202306002_SourceDataFS1.pdf]

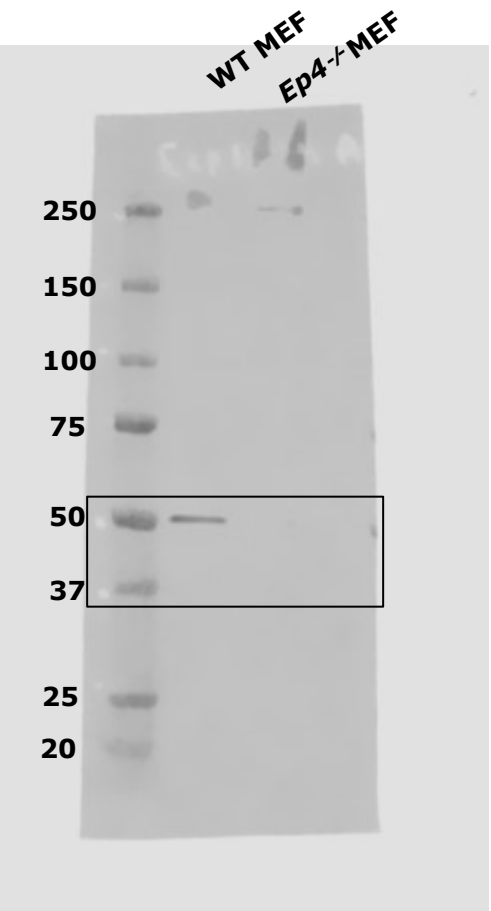

EP<sub>4</sub>

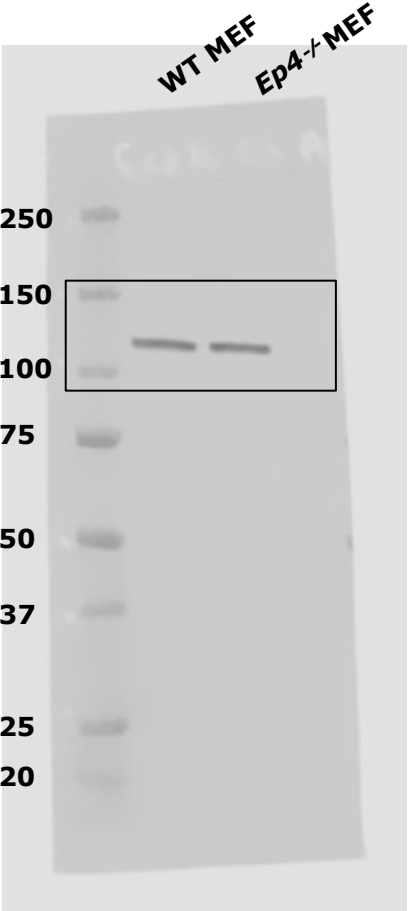

KINESIN

Supplement: SourceData FS2 — is the source file for Fig. S2. [file JCB_202306002_SourceDataFS2.pdf]

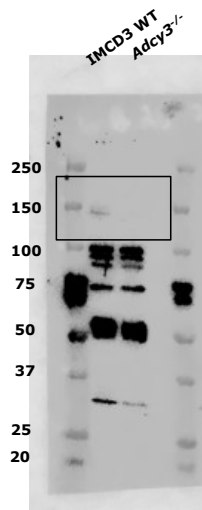

AC3

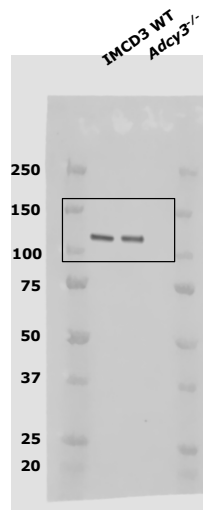

KINESIN

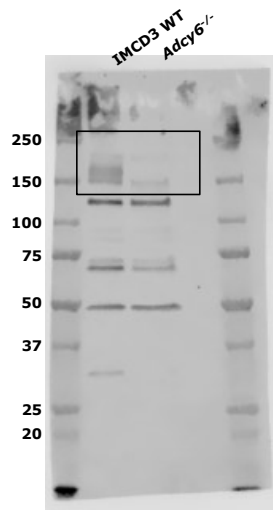

AC6

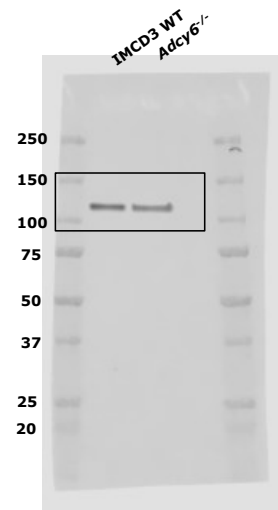

KINESIN

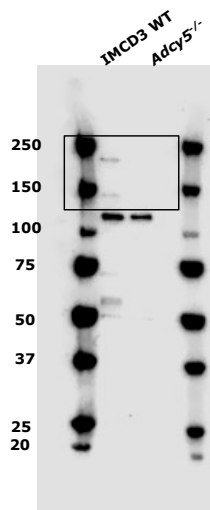

AC5

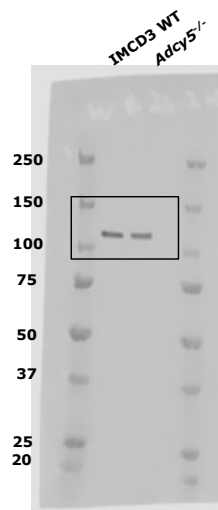

KINESIN

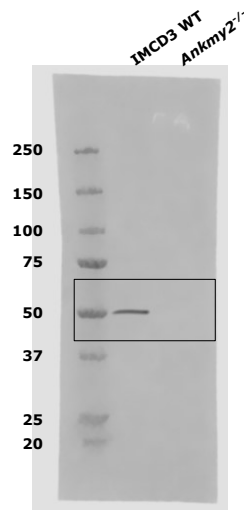

ANKMY2

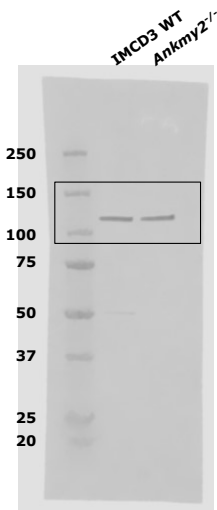

KINESIN

Supplement: SourceData FS3 — is the source file for Fig. S3. [file JCB_202306002_SourceDataFS3.pdf]
